# Effectiveness of a Person-Centered Interdisciplinary Rehabilitation Treatment of Post–COVID-19 Condition: Protocol for a Single-Case Experimental Design Study

**DOI:** 10.2196/63951

**Published:** 2024-10-11

**Authors:** Carolina M H Wiertz, Thijs van Meulenbroek, Cynthia Lamper, Bea Hemmen, Simone Sep, Ivan Huijnen, Marielle E J B Goossens, Jako Burgers, Jeanine Verbunt

**Affiliations:** 1 Department of Rehabilitation Medicine Care and Public Health Research Institute Maastricht University Maastricht Netherlands; 2 Adelante Centre of Expertise in Rehabilitation and Audiology Hoensbroek Netherlands; 3 Department of Rehabilitation Medicine Zuyderland Heerlen Netherlands; 4 Research Centre for Assistive Technology in Care Zuyd University of Applied Sciences Heerlen Netherlands; 5 Department of Family Medicine Care and Public Health Research Institute Maastricht University Maastricht Netherlands

**Keywords:** rehabilitation medicine, postacute COVID-19 syndrome, quality of life, long COVID, COVID-19, multidisciplinary care, interdisciplinary care

## Abstract

**Background:**

Patients with post–COVID-19 condition (PCC) experience a wide range of complaints (physical, cognitive, and mental), sometimes with high levels of disability in daily activities. Evidence of effective interdisciplinary rehabilitation treatment is lacking. A person-centered, biopsychosocial, interdisciplinary rehabilitation program, adapted to expert opinions and the patient’s needs, was therefore developed.

**Objective:**

This study aims to present a study protocol for a clinical trial to evaluate the effect of a new, person-centered, interdisciplinary rehabilitation treatment for PCC. It is aimed at improving participation in society and health-related quality of life in patients with PCC who perceive a high level of disability in daily activities or participation.

**Methods:**

A total of 20 Dutch adults, aged 18 years or older, with high levels of disability in daily activities and participation in society will be included in this replicated and randomized single-case experimental design study, from October 2023 onward. The replicated and randomized single-case experimental design consists of 3 phases. The baseline phase is the observational period, in which no specific treatment will be given. In the intervention phase, patients will receive the new outpatient treatment 3 times a week for 12 weeks, followed by a 12-week follow-up phase. During the intervention phase, the treatment will be personalized according to the patient’s physical, mental, and cognitive symptoms and goals. The treatment team can consist of a rehabilitation physician, physiotherapist, occupational therapist, speech therapist, and psychologist. The primary outcomes of the study are daily diaries, which consist of 8 questions selected from validated questionnaires (Utrecht Scale for Evaluation of Rehabilitation-Participation, EQ-5D-5L, and the Hospital Anxiety and Depression Scale). The other primary outcome measurements are participation in society (Utrecht Scale for Evaluation of Rehabilitation-Participation) and health-related quality of life (EQ-5D-5L). The secondary outcomes are physical tests and validated questionnaires aimed at physical, mental, and cognitive complaints. Effect evaluation based on daily assessments will include visual analysis, calculation of effect sizes (Nonoverlap of All Pairs), randomization tests, and multilevel analysis. In addition, other analyses will be based on ANOVA or a 2-tailed Student *t* test.

**Results:**

Data collection for this study started in October 2023 and is planned to be completed in July 2024. The results will be published in peer-reviewed journals and presented at international conferences.

**Conclusions:**

This is the first study investigating the effect of an interdisciplinary rehabilitation treatment with a person-centered, biopsychosocial approach in patients with PCC. Our findings will help to improve the treatment and support of patients with PCC.

**Trial Registration:**

German Clinical Trials Register DRKS00032636; https://drks.de/search/en/trial/DRKS00032636

**International Registered Report Identifier (IRRID):**

DERR1-10.2196/63951

## Introduction

A COVID-19 infection, caused by the SARS-CoV-2 virus, typically causes respiratory tract illness [1]. In the Netherlands, there have been more than 8.5 million COVID-19 infections since December 2019 [2]. Although most patients with COVID-19 fully recover, about 10% to 20% of them retain symptoms after recovery from the initial SARS-CoV-2 infection [1]. Post–COVID-19 condition (PCC), commonly known as long COVID, is defined as the continuation or development of new symptoms 3 months after the initial SARS-CoV-2 infection, with these otherwise unexplained symptoms lasting for at least 2 months [3]. PCC can affect anyone exposed to the SARS-CoV-2 virus, regardless of their age or the severity of the original symptoms. Although the exact number of patients with PCC is uncertain, it is believed that more than 1.7 million people across Europe may have experienced it during the first 2 years of the pandemic (2020-2021) [3]. Common symptoms of PCC include fatigue, dyspnea, anosmia, muscle aches, headache, chest pain, palpitations, cognitive dysfunction, anxiety or depression, and sleep problems [4,5].

Persistent symptoms associated with PCC seem to harm physical and cognitive function, health-related quality of life (HRQoL), and participation in society [4,6]. According to the vision of health by the World Health Organization (WHO)—as presented based on the *International Classification of Functioning, Disability and Health*—functional impairments as a result of biomedical and psychosocial factors can reduce social participation, quality of life, and the ability to perform activities [7]. Therefore, it is possible that persistent complaints of PCC can be maintained by both (defined or still undefined) biomedical and psychosocial factors. This is consistent with findings from one of our earlier prospective cohort studies, in which survivors of COVID-19 in intensive care units (ICUs) who were admitted due to severe biomedical complications in this early phase of their illness were followed over time. Patients’ levels of participation improved during the first year after ICU discharge [8]. But we also found that personal factors, such as less proactive coping, anxiety, or depression assessed 1 month after ICU discharge, appeared more predictive than biomedical (ICU-related) factors in indicating a delayed return to the patient’s habitual level of participation during the first year. These findings suggest that rehabilitation treatment for survivors of COVID-19 in ICUs should focus on strengthening personal and environmental factors to improve functioning, participation in society, and quality of life and not just on biomedical complaints. Although these findings were specifically related to survivors of COVID-19 in ICUs [8], the same treatment components may also apply to patients with an initially milder course of COVID-19 infection who go on to develop PCC. This would be in line with the advice of the WHO, which recommends that rehabilitation treatment should have a human-centered, goal-oriented, and holistic approach designed to optimize functioning in individuals with health conditions or impairments in interaction with their environment [9]. Guidelines from the WHO and others recommend an interdisciplinary rehabilitation treatment for long-lasting complaints due to PCC with a high level of disability [9-11].

Recently, multiple studies on the effect of rehabilitation treatment in PCC were published. Two systematic reviews show an uncertain-to-positive effect of physical (pulmonary) training on functional exercise capacity, dyspnea, and quality of life [12,13]. Neither systematic review found any serious adverse events for any of the physical (pulmonary) training [12,13]. In addition, 2 experimental studies found no postexertional malaise or other adverse events due to physical activity [14,15]. However, a recent study showed changes in skeletal muscle structure in patients with PCC that are associated with a lower exercise capacity [16]. Another systematic review showed that a multidisciplinary rehabilitation treatment (multiprofessional, programs were ambulatory or inpatient) may have beneficial effects on fatigue, dyspnea, physical capacity, pulmonary function, quality of life, and daily life activities [17]. However, this evidence is very uncertain due to the low level of evidence, inconsistency in the results, varying treatment programs, and varying patient characteristics. Based on the biopsychosocial focus of PCC, psychological interventions such as cognitive behavioral therapy (CBT) or acceptance and commitment therapy (ACT) could also be useful. These would be aimed at increasing psychological flexibility or strengthening a person’s resilience, depending on the patient’s problem [18,19]. In a study by Kuul et al [20], CBT was effective in reducing fatigue complaints and improving physical and social functioning in patients with PCC. Furthermore, CBT seemed to be both feasible and accepted by patients with PCC [21]. In addition, in a study of Nikrah et al [22], ACT significantly increased the resilience and HRQoL of patients with PCC. In summary, although there has been research on rehabilitation treatments for PCC, the current understanding of the most effective rehabilitation treatment approach and the effects on participation levels and HRQoL is still limited.

The precise cause of PCC remains unclear. A recent study by Appelman et al [16] showed changes in skeletal muscle structure, with severe exercise-induced myopathy and tissue infiltration of amyloid-containing deposits in the skeletal muscles of patients with PCC. Although there may be a somatic cause for PCC, a biopsychosocial approach seems promising for improving functioning in patients with PCC who perceive a high level of disability in daily activities and participation. It seems to be important to improve physical as well as mental resilience in order to optimize participation in society, despite health complaints due to PCC, by using a person-centered program focused on the various biopsychosocial factors of the patient and supported by a treatment plan from the entire interdisciplinary rehabilitation team. We therefore developed a new person-centered and tailored interdisciplinary rehabilitation treatment for patients with multiple health-related PCC complaints and high levels of disability in daily activities and participation. The treatment program is based on patients’ and health care professionals’ experiences and needs and on recommendations from the *European Physical and Rehabilitation Medicine* position paper [23]. This study aims to describe the replicated and randomized single-case experimental design (R-SCED) that is used to test the effectiveness of a 12-week, personalized, interdisciplinary rehabilitation treatment in secondary care and to evaluate changes in the recovery of participation levels and quality of life in patients with PCC.

## Methods

### Study Design

**Figure 1:**
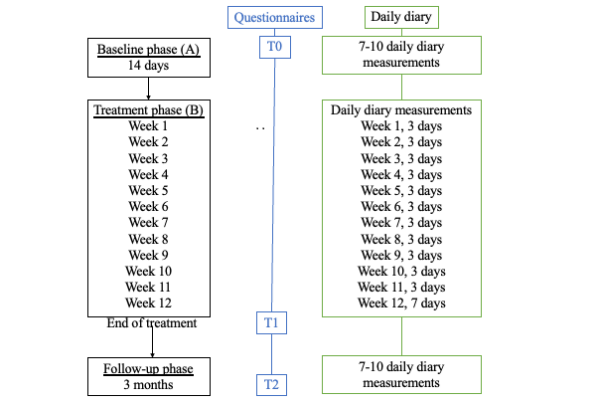
Schematic presentation of the design: (left) the R-SCED design and treatment and (right) measurements. R-SCED: replicated and randomized-single case experimental design; T0: beginning of the baseline phase (phase A); T1: after completion of the treatment phase (phase B); T2: after completion of the follow-up phase.

The design is R-SCED. This R-SCED study will use repeated measurements according to an AB follow-up design. In this design, patient functioning is closely monitored during a baseline phase (A), a treatment phase (B), and a follow-up phase. The length of phase A is 2 weeks. Phase B lasts for 12 weeks for all participants, and the follow-up phase lasts for 12 weeks. Figure 1 is a schematic presentation of the design, with treatment design and measurements (T0: beginning of phase A; T1: after completion of phase B; and T2: after completion of the follow-up phase). This R-SCED will be performed at Adelante Zorggroep, location MUMC+, which is an outpatient rehabilitation center in the south of the Netherlands.

### Ethical Considerations

The institutional review board of Maastricht University Medical Centre, University of Maastricht, approved this study (NL83848.068.23 or METC 23-008) in July 2023. All participants provided written informed consent. All personal data will be handled in accordance with the EU General Data Protection Regulation and the Dutch Act on Implementation of the General Data Protection Regulation. (in Dutch: Uitvoeringswet AVG, UAVG). Participating patients will receive €75,00 after finishing the diaries and all the questionnaires.

### Eligibility Criteria

Adults, aged 18 years or older, having an indication for outpatient interdisciplinary rehabilitation treatment for PCC (high levels of disability in daily activities and participation based on the expert opinion of the rehabilitation physician) and with adequate Dutch literacy were eligible for inclusion. Participants will be excluded if PCC symptoms could be explained by an alternative medical disease or if they are not able to speak or read the Dutch language fluently; have no access to a smartphone, tablet, or computer; or have insufficient digital skills.

### Regular Health Care Procedure

After referral by a general practitioner or medical specialist, the rehabilitation physician will invite the participant for a first consultation. This first consultation is for validating PCC complaints and their impact on daily life activities and participation. Biopsychosocial disabling factors will be identified and possibilities for psychological flexibility will be assessed. There will also be a medical examination, and other medical diseases will be excluded. If necessary, a referral to other medical specialists in secondary care will take place. Finally, education about PCC complaints and biopsychosocial factors for the participant and partner will be given [24]. During this consultation, a decision is made on whether there is an indication for an interdisciplinary rehabilitation treatment in secondary care. If an interdisciplinary rehabilitation treatment in secondary care is indicated and the participant meets the inclusion criteria, the rehabilitation physician will include the participant for the treatment and inform them about the aim and content of the study. Potential participants will have 1 week to reflect before deciding they want to participate. If the participant is willing to participate in the study, the study treatment protocol will start.

### Baseline Phase (Phase A)

The baseline phase (phase A) is the control period of each participant. No specific treatment will be given during this period. The start of phase A is determined based on the start date of the interdisciplinary rehabilitation treatment and lasts 2 weeks. The number of days (7, 8, 9, or 10) a participant must fill in a daily diary during phase A will be randomized by a dedicated computer program (YourResearch). Phase B will start directly after phase A.

### Treatment Phase (Phase B)

All participants will receive an outpatient interdisciplinary rehabilitation treatment in secondary care. This treatment aims to increase patients’ levels of activity, participation in daily life, and HRQoL regardless of the level of complaints they experience. During phase B, participants are invited to fill in a daily diary for 40 days (Figure 1).

The treatment team consists of a rehabilitation physician, physiotherapist, occupational therapist, speech therapist, and psychologist. The team is experienced in interdisciplinary rehabilitation treatment with a biopsychosocial approach. Education has been given and supervision team meetings are planned monthly. For each participant, the content of the interdisciplinary rehabilitation treatment will be personalized according to the participant’s individual physical, mental, and cognitive complaints and personal goals. As a result, input from the various disciplines can vary according to the participant’s problem profile and needs. The individual goals are activity- and participation-oriented and determined during the intake with the rehabilitation physician and further specified during the treatment (eg, “being able to vacuum the entire house”). The Canadian Occupational Performance Measure (COPM), administered by an occupational therapist, will be used to support individual goal setting. The COPM was developed to set treatment goals based on problems experienced by the patient in daily activities [25,26]. The rehabilitation physician is the team supervisor and has final medical responsibility for the participants during the study period.

The interdisciplinary rehabilitation treatment lasts 12 weeks, with the patient receiving therapy approximately 3 half days per week (depending on their goals and initial capacity). In order to improve the level of functioning, the first 4 weeks consist of an assessment phase, in which the various disciplines gain insight into the participant’s complaints, functional capabilities, and the potential to strengthen physical and mental resilience. After this first phase, a multidisciplinary consultation (MDC) takes place with all disciplines involved. During this MDC, an individual model explaining the level of COVID-19–related disability is proposed, based on the interaction of the disabling factors involved. The team will propose an 8-week treatment plan tailored to the patient’s individual problems and goals. After this MDC, the patient will receive an interdisciplinary education from the treatment team in which the proposed individual explanatory model from a biopsychosocial perspective is discussed with the participant, including the hypothesized disabling factors that interfere with performing activities and participation in daily life. This education with a biopsychosocial approach is aimed at increasing mental and physical resilience despite the PCC complaints. Another MDC will take place in weeks 7 and 11 to discuss the progress in the treatment process and whether treatment goals or treatment plans should be adjusted. After each MDC, the rehabilitation physician provides all feedback to the participant and discusses the proposed plan according to principles of shared decision-making. If necessary, the rehabilitation physician will give additional biopsychosocial education. There will be a follow-up consultation with the rehabilitation physician after 3 months of completing the treatment phase.

The following are the commitment of disciplines within the interdisciplinary rehabilitation treatment and the contribution of the disciplines can vary according to the patient’s profile and needs.

- Rehabilitation physician, at least 4 times (45 minutes for the first consultation, 15 minutes for a regular consultation): first consultation, after the MDC, and follow-up consultations.
- Physiotherapy, 2 times per week (each session takes 60 minutes), for improving physical performance such as physical condition and muscle strength through functional training and hydrotherapy and to guide in case of any pain- or tension-related complaints [11,27]. Patients are also encouraged to experience movement possibilities and will be encouraged to be as active as possible, regardless of the number and severity of complaints, particularly for valuable activities and social participation.
- Occupational therapy, 2 times per week (each session takes 60 minutes), for education in energy management and recognizing and pushing boundaries. The occupational therapist helps in resuming and building up daily activities (such as household duties, leisure activities, or work) despite the level of complaints [11,28].
- Speech therapy, 2 times per week (each session takes 30 minutes), for breathing exercises in case of dyspnea complaints, dysfunctional breathing, or dysfunction of the diaphragm [11].
- Psychotherapy, once a week (each session takes 60 minutes). To strengthen a person’s resilience, first and second-generation CBT as well as third-generation CBT (ACT) will be applied. CBT aims to improve quality of life by strengthening individual resilience to cope with complaints [19,29]. The treatment can include a variety of techniques such as psychoeducation, verbal challenge techniques (learning to critically examine dysfunctional thoughts), behavioral experiments, skills training, breathing and relaxation exercises, stress management, problem-solving skills, and exercises to broaden sensory perception. The goal of ACT is growth in psychological flexibility [18]. The emphasis here is not on controlling and changing, but on accepting thoughts, behavior, feelings, and experiences such as negative physical experiences, fatigue, and pain.

### Follow-Up Phase

The follow-up phase takes 12 weeks and starts directly after phase B. In the last 2 weeks of the follow-up phase, participants are invited to fill in a daily diary. The number of days (7,8, 9, or 10) a participant must fill in a daily diary during the last 2 weeks of the follow-up phase, will be randomized by a dedicated computer program (YourResearch). Phase B will start directly after phase A.

### Assessment

Since PCC can affect different domains of functioning, measurements were chosen based on an integral vision of health and include body function impairments, activity limitations, and participation restrictions as well as personal factors, according to the *International Classification of Functioning, Disability and Health* [7]. All variables will be measured by daily diary items, validated questionnaires, and physical examination (Figure 1 and Tables 1-3). Based on design standards, the recommended minimum number of baseline measurements in an R-SCED study is 5 [30]. In this study, daily diaries will be completed for 7 to 10 days (baseline measurements) in phase A. In addition, there are 40 measurements during the rehabilitation treatment and a minimum of 7 to 10 follow-up measurements. The validated self-administered questionnaires will be collected at the start of phase A (T0), at the end of phase B (T1), and after 3 months of follow-up (T2). Participants will complete the questionnaires and the daily measurements with an app (YourResearch) on a computer or mobile device.

**Table 1:** Overview of the secondary outcome measurements: body function.

Secondary outcome measurements	Measurement instrument	T0^a^	T1^b^	T2^c^
Muscle strength	Muscle strength is a commonly used test for physical function [31]. A Jamar HHD^d^ is used for handgrip muscle strength [32,33]. Muscle strength of the knee extensor or flexor is measured using a dynamometer (Biodex System 3 Pro Dynamometer) [34].	✓	✓	
Fatigue	MFIS^e^: this is a 21-item metric for fatigue severity. A total score is calculated as the sum of the subscale scores (0-84). Higher scores indicate higher levels of fatigue [35].	✓	✓	✓
Breathlessness	MRC^f^ breathlessness scale: ranging from 1=no trouble with breathlessness to 5=I am too breathless to leave the house [36].	✓	✓	✓
Pain	NRS^g^: mean pain intensities in the last 7 days, ranging from 0 to 10 [37].	✓	✓	✓
Cognitive function	CFQ^h^: this is a 25-item self-report questionnaire. The total scores range from 0 to 100, with higher scores reflecting a report of more frequent cognitive errors in everyday life [38,39].	✓	✓	✓

^a^T0: beginning of baseline phase (phase A).

^b^T1: after completion of the treatment phase (phase B).

^c^T2: after completion of the follow-up phase.

^d^HHD: handheld dynamometer.

^e^MFIS: Modified Fatigue Impact Scale.

^f^MRC: Medical Research Council.

^g^NRS: Numerical Rating Scale.

^h^CFQ: Cognitive Failure Questionnaire.

### Outcome Measurement

#### Primary Outcome Variables

The primary outcome measurements are participation in society and HRQoL. A daily diary will be used, including a short (10-item) questionnaire to assess changes over time. The daily diaries consist of 8 questions selected from the validated questionnaires: Utrecht Scale for Evaluation of Rehabilitation-Participation (USER-P), EQ-5D-5L, and the Hospital Anxiety and Depression Scale (Multimedia Appendix 1). Each question is rated on a scale from 0 to 10. In addition, the daily dairy will focus on 2 most important individual goals (the participant will be asked to rate each goal on performance and satisfaction, on a scale from 0 to 10, based on the COPM score). The individual goals are determined by the patient, immediately after inclusion in the study.

Validated self-administered questionnaires will also be used. Participation in society will be assessed by USER-P. The USER-P consists of 3 subscales: the frequency of activities, the restriction subscale, and the satisfaction subscale. For all 3 subscales, the total score ranges from 0 to 100, with higher scores indicating better participation [40,41]. HRQoL is evaluated with the 5-dimension descriptive system (EQ-5D-5L) and the associated visual analog scale [42]. The EQ-5D-5L includes 5 domains, namely, mobility, self-care, usual activities, pain, and anxiety or depression. Each dimension is rated on a 5-level scale from “no problems” to “unable.” For the resulting 5-digit EQ-5D health profile, a weighted EQ index was calculated using Dutch reference values [43]. The EQ-5D-5L visual analog scale asks the participant to value their perceived health from 0=the worst imaginable health to 100=the best imaginable health, with higher scores indicating higher quality of life.

#### Secondary Outcome Variables

Secondary outcome variables are presented in Tables 1-3. Body function impairments (muscle strength, fatigue, breathlessness, pain, cognitive impairments), activity limitations (functional exercise, perceived limitations in daily life), participation (individual goals, activity and participation oriented), and personal factors (baseline characteristics, anxiety, depression, coping style and resilience) are assessed combined with physical examination and validated self-administered questionnaires. Details of the measurement instruments are also presented in Tables 1-3.

**Table 2:** Overview of the secondary outcome measurements: activity limitations and participation.

Secondary outcome measurements	Measurement instrument	T0^a^	T1^b^	T2^c^
Functional exercise	6MWT^d^: a submaximal exercise capacity by walking distance. The distance reached will be recorded in meters [44]. STS^e^: for functional mobility, balance, and lower limb strength. Time in seconds is the main outcome [45,46]. The 2-minute SCT^f^: a submaximal exercise capacity. The number of steps is the main outcome [47]. The 6MWT, STS, and SCT will be performed by an experienced physiotherapist.	✓	✓	
Perceived limitations in daily life	PROMIS^g^ physical function short form 8b: has 8 questions. Higher scores indicate better physical function [48,49]	✓	✓	✓
Individual goals, activity, and participation oriented	COPM^h^: to set treatment goals based on problems experienced by the patient in daily activities and will be administered by an occupational therapist [25,26].	✓	✓	

^a^T0: beginning of baseline phase (A).

^b^T1: after completion of the treatment phase (B).

^c^T2: after completion of the follow-up phase.

^d^6MWT: 6-minute walk test.

^e^STS: Sit-to-stand test.

^f^SCT: stair climbing test.

^g^PROMIS: patient-reported outcomes measurement information system.

^h^COPM: Canadian Occupational Performance Measure.

**Table 3:** Overview of the secondary outcome measurements: personal factors.

Secondary outcome measurements	Measurement instrument	T0^a^	T1^b^	T2^c^
Baseline characteristics	Age, sex, weight, education level, and marital status, as well as information about the time of COVID-19 infection, current complaints since the COVID-19 infection, the need for hospitalization due to COVID-19 infection, who diagnosed the post–COVID-19 condition, and previous treatments for the post–COVID-19 condition (open question and participant opinion).	✓		
Anxiety and depression	HADS^d^: this is a 14-item self-rated scale. The total score ranges from 0 to 21. A score ≥8 on either subscale is considered to be substantial anxiety or depression symptoms [50].	✓	✓	✓
Proactive coping skills	UPCC^e^: a 21-item self-assessment tool. The total score was the average for all item scores (range 1-4), higher scores indicate higher levels of proactive coping [51].	✓		
Resilience	BRS^f^: a 6-item questionnaire. The total score is the mean score of all answers (range 1-5). Higher scores indicate a better-developed ability of resilience [52,53]	✓		

^a^T0: beginning of baseline phase (phase A).

^b^T1: after completion of the treatment phase (phase B).

^c^T2: after completion of the follow-up phase.

^d^HADS: Hospital Anxiety and Depression Scale.

^e^UPCC: Utrecht Proactive Coping Scale.

^f^BRS: Brief Resilience Scale.

### Sample Size Calculation

Experience from previous single-case experimental design (SCED) studies showed that the sample size varies between 6 and 27 participants [54-56]. One of our earlier prospective cohort studies was used in calculating the sample size. In this study, the USER-P was the primary outcome and improved in the first year after ICU discharge, [8]. In this cohort, the effect size based on changes in the level of participation appeared to be medium from baseline to 12 months follow-up (>0.5), with a statistical power of >0.8. Using the calculation tool developed by Bouwmeester and Jongerling [57] for SCED studies, with a medium effect size, the number of measurements, and accounting for loss-to-follow-up, 20 patients need to be included in this study. Patients withdrawn from the study will not be replaced. All analysis will be performed according to the “intention-to-treat” principle.

### Statistical Analysis

Baseline and demographic characteristics will be presented in means (SD), medians (IQR), or percentages, as appropriate.

For our primary outcome measurements, the daily dairies, the statistical analysis will be performed using the Shiny app (KU Leuven) for single-case data analysis [58]. The following steps will be taken to test the effects of the intervention. First, in visual analysis, the within-case data of the daily dairies will be plotted and visually inspected per participant with respect to within-phase (eg, level, trend, variability) and between-phase characteristics (eg, immediacy of effect, overlap, consistency) [59]. Second, the percentage of nonoverlapping pairs between phases will be used to calculate the effect size of each participant. Nonoverlapping data between phases indicate performance differences between phases and is an important part of visual analysis in single-case research [60]. The Nonoverlap of All Pairs is an index of data overlap between phases in single-case research [60]. The Nonoverlap of All Pairs equals the number of comparison pairs showing no overlap, divided by the total number of comparisons. It can be calculated as an area under the curve [60]. Third, randomization tests will be performed to test the null hypothesis; that the interdisciplinary rehabilitation treatment has no effect on participation levels and HRQoL. The observations of the baseline phase will be compared to those of the intervention and follow-up phases, respectively (*mean phase A – mean phase BC = test statistic*, where C is the follow-up phase). Because the starting point of phase B (intervention) is randomly determined and the follow-up phase does not include any new intervention methods, phase B and the follow-up phase are combined. In case visual analysis suggests delayed effects, the randomization test will be repeated with lagged data until the lowest *P* value has been reached. Fourth, in a SCED study, 1 outcome is repeatedly measured in the same person. These replicated single-case experiments may be considered as multiple studies that can be combined using meta-analytical procedures [61,62]. To evaluate the overall changes in the daily diaries the daily data in phase A, phase B, and follow-up a multilevel analysis will be used to summarize the single-case data. Previously calculated effect sizes will be combined.

Missing data will be handled according to the randomized-marker method, in which all days of the diary that were not completed will be displayed as “N/A” (not applicable) [63].

For the other primary outcome measurements (USER-P and EQ-5D-5L) and the secondary outcome measurements, the results on T0, T1, and T2 will be computed as total scores on each questionnaire individually. Outcomes will be presented in mean (SD) or median (IQR), depending on the distribution. Outliers will be checked, defined as values more than 3 times the IQR from the median. The statistical analysis of the questionnaires will use repeated measures ANOVA in case of normal distribution or the Friedman test in case of nonnormal distribution. The statistical analysis of the physical examination will use the paired 2-tailed Student *t* test in case of normally distributed variables or the paired Wilcoxon test in case of nonnormally distributed variables. The significance level was set at *P*=.05, 95% CI. The statistical analysis will use IBM SPSS statistics version 26. No imputation will be made for missing data.

### Data and Safety Monitoring

The risks attached to participating in this study are negligible and, in any case, no greater compared with other interdisciplinary rehabilitation treatments. A data safety and monitoring board has been appointed from de Clinical Trial Center Maastricht, with expertise in clinical trials. Monitoring includes informed consent forms, source data verification, and serious adverse events.

## Results

This study was funded in June 2022. The institutional review board of Maastricht University Medical Centre, University of Maastricht, approved this study in July 2023. Participant recruitment started in October 2023 and was completed in July 2024. Data collection for this study is expected to be completed by January 2025. The analysis will be conducted after data collection has been completed. It is expected that the results will be published in spring 2025.

## Discussion

### Expected Findings

This study will assess the effectiveness of a new person-oriented interdisciplinary rehabilitation treatment in patients with PCC.

The WHO and other international guidelines recommend an interdisciplinary rehabilitation treatment for long-lasting complaints due to PCC with high level of disability [9-11,23]. Although the number of studies evaluating (interdisciplinary) rehabilitation treatments for PCC has increased [12,13,17], knowledge regarding the effect of these rehabilitation treatments on participation levels and HRQoL is still limited. It is expected that this personalized interdisciplinary rehabilitation treatment for patients with PCC, who perceive high levels of disability in daily activities and participation, will improve their level of participation and HRQoL and that physical, mental, and cognitive complaints will decrease.

Next, it is expected that this interdisciplinary rehabilitation treatment, when proven effective, can be implemented in other rehabilitation centers in the Netherlands and beyond. At the same time, we anticipate that patients and health care professionals will be satisfied with the interdisciplinary rehabilitation treatment. Depending on the results of the study, the interdisciplinary rehabilitation treatment can be further improved, based on information derived from a study of the experiences of patients and health care professionals. In addition, further research is necessary regarding the cost-effectiveness of the treatment, and when proven effective, further implementation studies will be facilitated to implement the program in current rehabilitation health care in the Netherlands and beyond. The results of the study will be published in peer-reviewed journals and presented at international conferences.

### Strengths and Limitations

One strength of the study is its interdisciplinary approach. Various interventions (among others, physical training, energy management, breathing techniques, and psychological interventions) that may be effective in the treatment of PCC have been combined into a single interdisciplinary rehabilitation treatment program. Each patient is treated with a biopsychosocial approach aimed at improving physical and mental resilience and optimizing participation in society. Patients with PCC can experience various complaints such as fatigue and physical, mental, and cognitive complaints [5,64]. Patients included in this study experience high levels of disability in daily activities and low levels of participation, but the combination of their complaints may differ. An interdisciplinary rehabilitation treatment with a biopsychosocial approach tailored to a patient’s complaints and goals, and which takes into account personal and contextual factors, therefore seems promising.

Another strength of the study is the chosen design: SCED [65]. Randomized controlled trials (RCTs) are seen as the gold standard for evaluating treatment effectiveness [66]. However, RCTs are time-consuming, and SCEDs seem to be a good alternative [67]. SCED studies are characterized by multiple measurements in a few participants, which allows a small sample size, whereas RCTs are based on a few measurement moments in many participants. Each patient functions as its own control and SCEDs are considered to be more compatible with clinical practice than an RCT [65,68]. A SCED can result in strong internal and external validity [65]. This study aims to evaluate the effect of a person-oriented interdisciplinary rehabilitation treatment, which makes a SCED most appropriate.

One limitation of the study is that a SCED may be resource intensive. The need for detailed observation and repeated measurements for a single participant can be demanding. A second limitation in using a SCED may be the generalizability of results. Since the focus is on a single participant or a small group, it may be hard to generalize the results to larger and more diverse populations. However, this interdisciplinary rehabilitation treatment was developed for a selected population, that is, patients with high levels of disability in daily activities and participation due to PCC. This selected group is a good fit for the design of a SCED study. Nevertheless, making conclusions about the effectiveness of the treatment in patients with less severe complaints needs further consideration. More research will be needed regarding the characteristics of patients who are more likely to benefit from the treatment.

### Conclusions

To our knowledge, this will be the first study to investigate the effect of an interdisciplinary rehabilitation treatment with a person-centered, biopsychosocial approach, to aim at improving social participation and HRQoL for patients with PCC. Our findings will help to improve the treatment and support of patients with PCC.

## Appendix

Multimedia Appendix 1 Daily diary, English translation.

Multimedia Appendix 2 Peer-Review version 1 (including feedback on patient information letters and other medical-ethical documents) from the medical-ethical review board of Maastricht University Medical Centre, University of Maastricht.

Multimedia Appendix 3 Peer-Review version 2 (including feedback on patient information letters and other medical-ethical documents) from the medical-ethical review board of Maastricht University Medical Centre, University of Maastricht.

## Abbreviations

ACT: acceptance and commitment therapy
CBT: cognitive behavioral therapy
COPM: Canadian Occupational Performance Measure
HRQoL: health-related quality of life
ICU: intensive care unit
MDC: multidisciplinary consultation
PCC: post–COVID-19 condition
RCT: randomized controlled trial
R-SCED: replicated and randomized single-case experimental design
SCED: single-case experimental design
USER-P: Utrecht Scale for Evaluation of Rehabilitation-Participation
WHO: World Health Organization

## Data Availability

The data sets generated or analyzed during this study are available from the corresponding author on reasonable request.

## References

[1] (2020). Coronavirus disease (COVID-19) pandemic. World Health Organization.

[2] (2023). COVID dashboard Nederland.

[3] (2022). Post COVID-19 condition (long COVID). World Health Organization.

[4] Malik P, Patel K, Pinto C, Jaiswal R, Tirupathi R, Pillai S, Patel U (2022). Post-acute COVID-19 syndrome (PCS) and health-related quality of life (HRQoL)—a systematic review and meta-analysis. J Med Virol.

[5] van Kessel SAM, Olde Hartman TC, Lucassen P, van Jaarsveld CHM (2022). Post-acute and long-COVID-19 symptoms in patients with mild diseases: a systematic review. Fam Pract.

[6] Tabacof L, Tosto-Mancuso J, Wood J, Cortes M, Kontorovich A, McCarthy D, Rizk D, Rozanski G, Breyman E, Nasr L, Kellner C, Herrera JE, Putrino D (2022). Post-acute COVID-19 syndrome negatively impacts physical function, cognitive function, health-related quality of life, and participation. Am J Phys Med Rehabil.

[7] (2001). International Classification of Functioning, Disability and Health (ICF). World Health Organization.

[8] Wiertz CMH, Hemmen B, Sep S, van Santen S, van Horn YY, van Kuijk SMJ, Verbunt JA (2022). Life after COVID-19: the road from intensive care back to living—a prospective cohort study. BMJ Open.

[9] (2023). Strengthening rehabilitation in health systems. World Health Organization.

[10] (2020). COVID-19 rapid guideline: managing the long-term effects of COVID-19. National Institute for Health and Care Excellence.

[11] (2022). COVID-19. Federatie Medisch Specialisten.

[12] Pouliopoulou DV, Macdermid JC, Saunders E, Peters S, Brunton L, Miller E, Quinn KL, Pereira TV, Bobos P (2023). Rehabilitation interventions for physical capacity and quality of life in adults with post-COVID-19 condition: a systematic review and meta-analysis. JAMA Netw Open.

[13] Pollini E, Lazzarini S, Cordani C, Del Furia MJ, Kiekens C, Negrini S, Arienti C (2024). Effectiveness of rehabilitation interventions on adults with COVID-19 and post-COVID-19 condition. A systematic review with meta-analysis. Arch Phys Med Rehabil.

[14] Kupferschmitt A, Langheim E, Tüter H, Etzrodt F, Loew T, Köllner V (2022). First results from post-COVID inpatient rehabilitation. Front Rehabil Sci.

[15] Frisk B, Jürgensen M, Espehaug B, Njøten KL, Søfteland E, Aarli B, Kvale G (2023). A safe and effective micro-choice based rehabilitation for patients with long COVID: results from a quasi-experimental study. Sci Rep.

[16] Appelman B, Charlton B, Goulding R, Kerkhoff T, Breedveld E, Noort W, Offringa C, Bloemers FW, van Weeghel M, Schomakers BV, Coelho P, Posthuma JJ, Aronica E, Joost Wiersinga W, van Vugt M, Wüst RC I (2024). Muscle abnormalities worsen after post-exertional malaise in long COVID. Nat Commun.

[17] Dillen H, Bekkering G, Gijsbers S, vande Weygaerde Y, van Herck M, Haesevoets S, Bos DAG, Li A, Janssens W, Gosselink R, Troosters T, Verbakel JY (2023). Clinical effectiveness of rehabilitation in ambulatory care for patients with persisting symptoms after COVID-19: a systematic review. BMC Infect Dis.

[18] Hayes SC (2019). Acceptance and commitment therapy: towards a unified model of behavior change. World Psychiatry.

[19] Price JR, Mitchell E, Tidy E, Hunot V (2008). Cognitive behaviour therapy for chronic fatigue syndrome in adults. Cochrane Database Syst Rev.

[20] Kuut TA, Müller F, Csorba I, Braamse A, Aldenkamp A, Appelman B, Assmann-Schuilwerve E, Geerlings SE, Gibney KB, Kanaan RAA, Mooij-Kalverda K, Olde Hartman TC, Pauëlsen D, Prins M, Slieker K, van Vugt M, Keijmel SP, Nieuwkerk P, Rovers CP, Knoop H (2023). Efficacy of cognitive-behavioral therapy targeting severe fatigue following coronavirus disease 2019: results of a randomized controlled trial. Clin Infect Dis.

[21] Huth D, Bräscher A-K, Tholl S, Fiess J, Birke G, Herrmann C, Jöbges M, Mier D, Witthöft M (2024). Cognitive-behavioral therapy for patients with post-COVID-19 condition (CBT-PCC): a feasibility trial. Psychol Med.

[22] Nikrah N, Bahari F, Shiri A (2023). Effectiveness of the acceptance and commitment therapy on resilience and quality of life in patients with post-acute COVID-19 syndrome. Appl Nurs Res.

[23] Ceravolo MG, Anwar F, Andrenelli E, Udensi C, Qureshi J, Sivan M, Kiekens C, Zampolini M (2023). Evidence-based position paper on physical and rehabilitation medicine professional practice for persons with COVID-19, including post COVID-19 condition: the European PRM position (UEMS PRM Section). Eur J Phys Rehabil Med.

[24] (2022). Long-term health complaints after COVID-19. NHG-standaard.

[25] Law M, Baptiste S, McColl M, Opzoomer A, Polatajko H, Pollock N (1990). The Canadian Occupational Performance Measure: an outcome measure for occupational therapy. Can J Occup Ther.

[26] Carswell A, McColl M, Baptiste S, Law M, Polatajko H, Pollock N (2004). The Canadian Occupational Performance Measure: a research and clinical literature review. Can J Occup Ther.

[27] Koninklijk Nederlands Genootschap voor Fysiotherapie (2022). KNGF position on Physiotherapy in COVID-19.

[28] Wassink Dorethé, van de Ven-Stevens Lucelle (2022). Guidelines for Occupational Therapy for Clients with Post-COVID Syndrome.

[29] de C Williams AC, Fisher E, Hearn L, Eccleston C (2020). Psychological therapies for the management of chronic pain (excluding headache) in adults. Cochrane Database Syst Rev.

[30] Kratochwill TR, Hitchcock JH, Horner RH, Levin JR, Odom SL, Rindskopf DM, Shadish WR (2012). Single-case intervention research design standards. Remedial Spec Educ.

[31] Bohannon RW (2009). Dynamometer measurements of grip and knee extension strength: Are they indicative of overall limb and trunk muscle strength?. Percept Mot Skills.

[32] Hamilton A, Balnave R, Adams R (1994). Grip strength testing reliability. J Hand Ther.

[33] Roberts HC, Denison H, Martin H, Patel H, Syddall H, Cooper C, Sayer AA (2011). A review of the measurement of grip strength in clinical and epidemiological studies: towards a standardised approach. Age Ageing.

[34] Drouin JM, Valovich-mcLeod TC, Shultz S, Gansneder B, Perrin D (2004). Reliability and validity of the Biodex system 3 pro isokinetic dynamometer velocity, torque and position measurements. Eur J Appl Physiol.

[35] Kos D, Kerckhofs E, Nagels G, D'Hooghe BD, Duquet W, Duportail M, Ketelaer P (2003). Assessing fatigue in multiple sclerosis: Dutch modified fatigue impact scale. Acta Neurol Belg.

[36] Stenton C (2008). The MRC breathlessness scale. Occup Med.

[37] Williamson A, Hoggart B (2005). Pain: a review of three commonly used pain rating scales. J Clin Nurs.

[38] Broadbent D E, Cooper P, FitzGerald P, Parkes K (1982). The Cognitive Failures Questionnaire (CFQ) and its correlates. Br J Clin Psychol.

[39] Bridger RS, Johnsen S, Brasher K (2013). Psychometric properties of the Cognitive Failures Questionnaire. Ergonomics.

[40] Post MWM, van der Zee CH, Hennink J, Schafrat C, Visser-Meily J, van Berlekom SB (2012). Validity of the Utrecht Scale for Evaluation of Rehabilitation-Participation. Disabil Rehabil.

[41] van der Zee CH, Visser-Meily J, Lindeman E, Jaap Kappelle L, Post M (2013). Participation in the chronic phase of stroke. Top Stroke Rehabil.

[42] Balestroni G, Bertolotti G (2012). EuroQol-5D (EQ-5D): an instrument for measuring quality of life. Monaldi Arch Chest Dis.

[43] M Versteegh Matthijs, M Vermeulen Karin, M A A Evers Silvia, de Wit G Ardine, Prenger Rilana, A Stolk Elly (2016). Dutch Tariff for the Five-Level Version of EQ-5D. Value Health.

[44] ATS Committee on Proficiency Standards for Clinical Pulmonary Function Laboratories (2002). ATS statement: guidelines for the six-minute walk test. Am J Respir Crit Care Med.

[45] Bohannon RW (2006). Reference values for the five-repetition sit-to-stand test: a descriptive meta-analysis of data from elders. Percept Mot Skills.

[46] Tiedemann A, Shimada H, Sherrington C, Murray S, Lord S (2008). The comparative ability of eight functional mobility tests for predicting falls in community-dwelling older people. Age Ageing.

[47] Harding VR, de C Williams AC, Richardson P, Nicholas M, Jackson J, Richardson I, Pither CE (1994). The development of a battery of measures for assessing physical functioning of chronic pain patients. Pain.

[48] Feng D, Laurel F, Castille D, McCormick AKHG, Held S (2020). Reliability, construct validity, and measurement invariance of the PROMIS physical function 8b-adult short form v2.0. Qual Life Res.

[49] HealthMeasures Scoring Service powered by Assessment CenterSM.

[50] Bjelland I, Dahl AA, Haug TT, Neckelmann D (2002). The validity of the Hospital Anxiety and Depression Scale. An updated literature review. J Psychosom Res.

[51] Bode C, Thoolen B, Ridder D (2008). Measuring proactive coping skills: psychometric properties of the Utrecht proactive Coping Competency List (UPCC). Psychologie Gezondheid.

[52] Smith BW, Dalen J, Wiggins K, Tooley E, Christopher P, Bernard J (2008). The brief resilience scale: assessing the ability to bounce back. Int J Behav Med.

[53] Leontjevas R, de Beek WO, Lataster J, Jacobs N (2014). Resilience to affective disorders: a comparative validation of two resilience scales. J Affect Disord.

[54] Leeuw M, Goossens MEJB, Linton SJ, Crombez G, Boersma K, Vlaeyen JWS (2007). The fear-avoidance model of musculoskeletal pain: current state of scientific evidence. J Behav Med.

[55] de Jong JR, Vlaeyen JWS, Onghena P, Goossens MEJB, Geilen M, Mulder H (2005). Fear of movement/(re)injury in chronic low back pain: education or exposure in vivo as mediator to fear reduction?. Clin J Pain.

[56] Vlaeyen JWS, de Jong J, Geilen M, Heuts P, van Breukelen G (2002). The treatment of fear of movement/(re)injury in chronic low back pain: further evidence on the effectiveness of exposure in vivo. Clin J Pain.

[57] Bouwmeester S, Jongerling J (2020). Power of a randomization test in a single case multiple baseline AB design. PLoS One.

[58] (2020). Shiny SCDA. Methodology of Educational Sciences Research Group of KU Leuven.

[59] Kratochwill TR, Levin JR (2014). Visual analysis of single-case intervention research: conceptual and methodological issues. Single-Case Intervention Research: Methodological and Statistical Advances.

[60] Parker RI, Vannest K (2009). An improved effect size for single-case research: Nonoverlap of All Pairs. Behav Ther.

[61] Onghena P, van Damme G (1994). SCRT 1.1: single-case randomization tests. Behav Res Methods Instrum Comput.

[62] Moeyaert M, Manolov R, Rodabaugh E (2020). Meta-analysis of single-case research via multilevel models: fundamental concepts and methodological considerations. Behav Modif.

[63] De TK, Michiels B, Tanious R, Onghena P (2020). Handling missing data in randomization tests for single-case experiments: a simulation study. Behav Res Methods.

[64] Lopez-Leon S, Wegman-Ostrosky T, Perelman C, Sepulveda R, Rebolledo P, Cuapio A, Villapol S (2021). More than 50 long-term effects of COVID-19: a systematic review and meta-analysis. Sci Rep.

[65] Lobo MA, Moeyaert M, Baraldi Cunha A, Babik I (2017). Single-case design, analysis, and quality assessment for intervention research. J Neurol Phys Ther.

[66] Hariton E, Locascio JJ (2018). Randomised controlled trials - the gold standard for effectiveness research: study design: randomised controlled trials. BJOG.

[67] Yang L, Armijo-Olivo S, Gross D (2023). Single-case experimental design in rehabilitation: basic concepts, advantages, and challenges. Am J Phys Med Rehabil.

[68] Onghena P, Edgington ES (2005). Customization of pain treatments: single-case design and analysis. Clin J Pain.

