# Supplementary material for: Effectiveness of a Person-Centered Interdisciplinary Rehabilitation Treatment of Post–COVID-19 Condition: Protocol for a Single-Case Experimental Design Study
Source: JMIR Res Protoc. 2024 Oct 11;13:e63951. doi: 10.2196/63951 (PMC11512124; doi:10.2196/63951)
Supplement: Multimedia Appendix 1 [file resprot_v13i1e63951_app1.pdf]

Appendix A; Daily diary

1. We would like to know how good or bad your health is today. This line is numbered from 0 to 10. Ten means the best health you can imagine and 0 the worst health you can imagine. Please mark the number on the line that shows you good or bad your health is today.
2. To what extent were you limited in daily life activities (such as: work, education, household duties) in the past 24 hours due to your COVID complaints? On the scale, '10' means not at all limited and '0' very limited.
3. To what extent were you limited in physical exercise (such as: walking, cycling, sport activities) in the past 24 hours due to your COVID complaints? On the scale, '10' means not at all limited and '0' very limited.
4. To what extent were you limited in leisure activities at home (such as: crafts, needlework, reading, puzzles, playing computer games) in the past 24 hours due to your COVID complaints? On the scale, '10' means not at all limited and '0' very limited.
5. To what extent were you limited in social contacts (such as: relationship with partner or going to visit family or friends) in the past 24 hours due to your COVID complaints? On the scale, '10' means not at all limited and '0' very limited.

The following statements are about how you feel today. On the scale, '10' means completely agree and '0' means completely disagree

6. I can do the things that I find important despite my COVID complaints (0-10).
7. I felt tense during the past 24 hours (0-10).
8. I felt cheerful during the past 24 hours (0-10)

Two personal goals

Activity 1: ……………………………………………………………………

a. To what extent were you able to perform this activity today? On the scale ‘10’ means totally able and ‘1’ not able to do at all.
b. How satisfied are you with the way you currently perform this activity today? On the scale ‘10’ means totally satisfied and ‘1’ not satisfied to do at all.

Activity 2: ……………………………………………………………………

a. To what extent were you able to perform this activity today? On the scale ‘10’ means totally able and ‘1’ not able to do at all.
b. How satisfied are you with the way you currently perform this activity today? On the scale ‘10’ means totally satisfied and ‘1’ not satisfied to do at all.
