# Supplementary material for: Effectiveness of a Person-Centered Interdisciplinary Rehabilitation Treatment of Post–COVID-19 Condition: Protocol for a Single-Case Experimental Design Study
Source: JMIR Res Protoc. 2024 Oct 11;13:e63951. doi: 10.2196/63951 (PMC11512124; doi:10.2196/63951)
Supplement: Multimedia Appendix 2 [file resprot_v13i1e63951_app2.pdf]

Dr. C. Lamper
Department of Rehabilitation Medicine
Maastricht University

ons kenmerk 23.0008725
doorkiesnummer 043-3876009
datum 09/05/2023

Betreft: Reactie n.a.v. bespreking in METC azM/UM - NL83848.068.23 / METC23-008
Titel: Effectiveness of an Interdisciplinary rehabilitation treatment for patients with post-COVID syndrome

Geachte dr. Lamper,

De medisch-ethische toetsingscommissie (METC) azM/UM heeft bovengenoemd onderzoeksvoorstel besproken in haar vergadering van 08-03-2023. Onze excuses voor de late terugkoppeling.

Het voorliggende onderzoeksvoorstel is op vele punten onvoldoende uitgewerkt en niet duidelijk. Hierdoor kan de commissie het onderzoeksvoorstel niet goed beoordelen. Zo is onder andere het wetenschappelijk belang van het onderzoek niet duidelijk; dat maakt dat bijv. niet beoordeeld kan worden of de risico's en belasting voor de studiedeelnemers opwegen tegen het belang van het onderzoek. Verder behoeft ook de opzet / het design van het onderzoek verdere verduidelijking. Ook lijkt de informatie over de opzet in de proefpersoneninformatiebrief niet overeen te komen met de informatie in het protocol. Onderstaand treft u een eerste overzicht van vragen en opmerkingen aan. Na ontvangst van uw reactie zal de commissie het onderzoeksvoorstel opnieuw integraal beoordelen in een van haar plenaire vergaderingen.

Voor een vlotte afhandeling wordt u gevraagd in uw reactiebrief de vragen van de commissie over te nemen en te laten volgen door uw inhoudelijk antwoord op de gestelde vragen, met daarbij (waar van toepassing) verwijzingen naar waar de aanpassingen in de documenten doorgevoerd zijn. Er kunnen alleen documenten in behandeling genomen worden waarin wijzigingen door middel van onderstreping zijn gemarkeerd (m.u.v. het ABR-formulier). Ieder document dient voorzien te zijn van een paginanummer en een versiedatum.

Belangrijkste vragen en opmerkingen

Met betrekking tot het onderzoeksprotocol:

1. Wetenschappelijk belang en achtergrond van het onderzoek:
   De voorliggende studie beoogt het effect te onderzoeken van een individuele interdisciplinaire tweedelijns revalidatiebehandeling bij patiënten met post-COVID syndroom die ernstig beperkt zijn in het dagelijks functioneren. Op dit terrein is al een aantal studies zowel uit de eerste als uit de tweede lijn verschenen. Wat voegt de voorliggende studie toe ten opzichte van het recent gepubliceerd onderzoek van Nopp (referentie 12 in het studieprotocol) en andere eerdere studies? U wordt gevraagd het wetenschappelijk belang van het voorliggende onderzoek verder te motiveren.
   - We danken de METC voor haar opmerkingen. Het klopt dat in de eerdere introductie tekst onvoldoende duidelijk wordt gemaakt waarin deze interdisciplinaire revalidatie behandeling anders is dan de behandelingen die reeds eerder zijn beschreven. De tekst in het protocol is daarom aangepast naar: '*Although these are positive results, both studies included only a small number of patients and most of them has been hospitalized for their COVID-19 infection. Therefore, these results cannot be generalized to the total PCS population. In addition, the treatments evaluated have no integral perspective on health complaints, participation and rehabilitation treatment. Without involvement of an occupational therapist, speech therapist and no or minimal involvement of a psychologist. Furthermore, more and more insights are emerging for a holistic approach, which considers various factors including biological, psychological, and social aspects, in the rehabilitation treatment of PCS (13,14). As well the World Health Organisation (WHO) as the NICE guideline recommends an interdisciplinary rehabilitation treatment for long-lasting complaints due to PCS (1,3)*'.
   - Daarbij maken beide studies gebruik van een pre-post design. Doordat wij gebruik maken van een gerandomiseerde experimentele studie is de bewijskracht ten aanzien van het effect van de interdisciplinaire revalidatie behandeling groter.

2. Hypothese, vraagstellingen en opzet van het onderzoek
   1) Hypothese is dat de deelnemers voordelen hebben van de interventie. De interventie (i.e. de interdisciplinaire revalidatiebehandeling in de tweede lijn) lijkt echter los te staan van het voorliggende onderzoek, daar de deelnemers geput worden uit de populatie die verwezen is voor een interdisciplinaire revalidatiebehandeling in de tweede lijn. De voorliggende studie beoogt het effect van de interdisciplinaire behandeling te meten.
      Ontvangen de studiedeelnemers reguliere zorg die intensief wordt opgevolgd middels vragenlijsten en interviews of wijkt de interdisciplinaire revalidatiebehandeling in de tweede lijn af van de standaardzorg? Verder: uit het protocol wordt niet duidelijk wanneer de hypothese wordt bevestigd. Wanneer is er sprake van het gewenste effect?
      - We danken de METC voor bovenstaande opmerking. In onze reactie op deze en onderstaande vragen willen we het protocol graag verder verduidelijken. Op dit moment is er nog geen standaard zorg voor post-COVID syndroom. Deze suggestie hebben we wel gewekt door de term 'care as usual' te gebruiken, zie ook het antwoord op onderstaande vraag. Patiënten krijgen een interdisciplinaire revalidatiebehandeling, waarin het effect van de behandeling wordt vergeleken met een baseline fase (A), zie

ook het antwoord op onderstaande vraag. Patiënten worden door huisarts of andere medisch specialist verwezen naar de revalidatiearts. Door de revalidatiearts wordt een beoordeling gemaakt of iemand in aanmerking komt voor een medisch specialistische revalidatie behandeling in de tweede lijn. Als de patiënt hiervoor in aanmerking komt, wordt gevraagd of diegene wil deelnemen aan het onderzoek. Indien de patiënt wil deelnemen, start fase A. In fase A wordt *geen* aanvullende behandeling gegeven vanuit de medisch specialistische revalidatie (baseline fase, geen interventie, variabel in tijd). De nieuwe interdisciplinaire revalidatie behandeling in de tweede lijn start pas in fase B. Het startmoment van fase B wordt bepaald op basis van loting (dit zorgt ervoor dat er sprake is van een gerandomiseerde studie (SCED)). Enkel deelnemers aan de SCED worden intensief opgevolgd met vragenlijsten en interviews.

- In het protocol stond geen specifieke hypothese beschreven. Deze is toegevoegd in hoofdstuk 2. Objectives. '*The hypothesis is that a personalized interdisciplinary rehabilitation treatment for patients with PCS will improve their participation levels and QoL, and will decrease physical, mental and cognitive complaints* '.

2) Primair doel is het evalueren van de effectiviteit van een 12 weken durende, gepersonaliseerde interdisciplinaire revalidatiebehandeling in de tweede lijn vergeleken met care as usual (standaardbehandeling).

- Het is volledig onduidelijk hoe de vergelijking met de standaardzorg gemaakt wordt. Er bestaan verschillende nazorgprogramma's. Is er consensus over wat usual care is? Wat houdt de usual care in? Aan wie wordt de usual care in de voorliggende studie gegeven en hoe wordt de uitkomst van de usual care gemeten? En hoe lang wordt de usual care gegeven: is dat ook 12 weken of is dat wat in de baseline fase wordt gegeven? Het lijkt er niet op dat de deelnemers in de tweede fase worden gerandomiseerd tussen de gepersonaliseerde behandeling en standaardzorg.
    - Momenteel is er nog geen standaard zorg binnen de revalidatie-geneeskunde voor de behandeling van post-COVID syndroom. De term 'care as usual' in de objectives is daarom ook onjuist gekozen in de eerste versie van het C1 protocol en heeft om die reden ook mogelijk tot verwarring geleid. Dit is ook reeds telefonisch al besproken met prof. Verbunt in mei 2023.
    - Met 'care as usual' werd bedoeld dat de interventie in fase B wordt vergeleken met een baseline fase (A) waarin geen behandeling zal plaatsvinden (geen revalidatiegeneeskundige behandeling, variabel in tijd). We snappen dat de term 'care as usual' onjuist gekozen is, waardoor het besluit is gemaakt om de primary objective te veranderen naar: '*To test the effectiveness of a 12-week personalized interdisciplinary rehabilitation treatment in secondary care and to evaluate changes in the recovery of participation levels and quality of life in patients with post-COVID syndrome as compared to a no-treatment randomized baseline periode (situation without rehabilitation treatment) and that treatment gains would be maintained at follow-up*'.

- Indien iemand wil deelnemen, start fase A. In fase A wordt geen revalidatiegeneeskundige behandeling gegeven en is variabel in tijd. Doel van deze opzet is om te onderzoeken of de nieuwe interdisciplinaire revalidatie behandeling tijdens behandeling (in fase B) en na behandeling (in fase C) effect heeft op de te meten uitkomsten ten opzichte van baseline (fase A) binnen proefpersonen. Door de SCED worden mensen vergeleken met zichzelf, we vergelijken de nieuwe interdisciplinaire revalidatiebehandeling met een situatie waarin geen revalidatiegeneeskundige behandeling wordt gegeven. Door de verschillende antwoorden en door de pprimary objective aan te passen hopen wij dan ook de verwarring weg te nemen. Aangezien veel van onderstaande vragen hier ook mee van doen hebben en mogelijk komen te vervallen met dit antwoord.

- U spreekt van een single case experimental design (SCED), maar dat lijkt niet correct. Er lijkt sprake van een eenvoudig pre-post design waarin eerst standaardzorg gegeven wordt (in fase A?) en daarna een gepersonaliseerd programma. Vraag is of je dan nog wel een goede vergelijking kunt maken. Immers: in welke mate kan standaardzorg de klachten/ beperkingen doen verminderen en in welke mate werkt dat door in de effectiviteit van de tweede fase waarin de gepersonaliseerde interventie wordt gegeven? Is een wash-out fase nuttig?
  - Zoals reeds aangegeven begrijpen we dat de verwarring is ontstaan over het design, gezien we spraken over de vergelijking met usual care. Er is wel degelijk sprake van een single case experimental design (SCED). Tijdens de baseline fase A vindt geen interventie plaats, er vinden slechts herhaalde metingen plaats. Doordat de duur van fase A (op basis van een gerandomiseerde duur van de baseline periode) bij iedere patiënt wisselt, kan uiteindelijk met meer zekerheid gezegd worden dat het gevonden verschil niet door verloop van tijd en spontaan beloop komt. In fase B vindt de revalidatiebehandeling plaats. Van een wash-out kan geen sprake zijn, omdat er altijd sprake zal zijn van een leereffect. Bij de revalidatiebehandeling hoop je dat er een onomkeerbaar effect zal plaatsvinden van de behandeling. Het doel van follow-up fase C is om te testen of het behandeleffect beklijft na 3 maanden follow-up.
  - Om het gehele design van de SCED te verduidelijken is de tekst in hoofdstuk 3. ‘*Study Design’* verder aangepast.
- Niet duidelijk is waarom er in fase A wordt gerandomiseerd. Fase A lijkt standaardzorg, of zit het toch anders in elkaar?
  - Zie bovenstaande antwoord. Tijdens fase A vindt geen interventie plaats. De duur van fase A bij iedere patiënt wisselt en daarmee het startmoment van de interventiefase (op basis van een randomisatie van de duur van de baseline fase). Er wordt dus gerandomiseerd op duur van de baseline fase en niet zoals in een RCT gerandomiseerd op behandeling. Wanneer tijdens fase A de klachten stabiel blijven, maar verbetering wel zichtbaar is in fase B (de behandelfase) dan kun je aannemen dat de verandering door de behandeling komt en niet door het verloop van de tijd.

3) Een van de secundaire doelen is het onderzoeken van de uitvoerbaarheid (feasibility) van de tweedelijns interdisciplinaire revalidatie behandeling. Het feasibility onderdeel is echter onvoldoende uitgewerkt in het voorliggende onderzoeksvoorstel.
   - In hoofdstuk 5. Treatment of subjects, is een extra toevoeging gedaan rondom de uitvoering van de feasibility studie. In hoofdstuk 6.2 Secondary study parameters/endpoints; wordt verwezen naar 2 verschillende bijlages waarin dieper wordt ingegaan op de inhoud van de focusgroepen en de patiënt interviews. Daarnaast wordt ook verwezen naar een eerdere METC-aanvraag (niet-WMO) vanuit hetzelfde onderzoeksproject, waarbij de focusgroepen met zorgverleners ook reeds in beschreven staan: MUMC, METC 2022-3439 "PINCOR”.
   - In hoofdstuk 5. Treatment of subjects is toegevoegd: ‘*After all included patients have completed the interdisciplinary rehabilitation treatment (phase B), a focus group lasting 1.5 hours will be organized with all disciplines of healthcare providers working with post-COVID patients within the interdisciplinary rehabilitation team (consultant in rehabilitation medicine, physiotherapists, occupational therapists, speech therapists and psychologists). The aim is to gather at least 5 healthcare providers and at least one from each discipline. Furthermore, all included patients will be interviewed at the end of the interdisciplinary rehabilitation treatment (phase B). The (semi-structured) interview will take place through video calling and will take about 30 minutes, or in a face-to-face- or online interview if the patient prefers this. All interviews will be recorded. The focus groups and patient interviews are described in MUMC, METC 2022-3439 "PINCOR”.*

3. In- en exclusiecriteria
   Het is van belang om mensen die voor de COVID-infectie al beperkt waren in dagelijkse activiteiten van deelname uit te sluiten. De exclusiecriteria graag op dit punt aanpassen.
   - We danken de METC voor deze waardevolle toevoeging. Aan de exclusie criteria is toegevoegd: ‘*Experiencing high levels of disability in daily activities and/or participation before their COVID-19 infection*.

4. Sample-size berekening en statistiek
   1) M.b.t. de sample-size:
   De sample-size berekening is vervangen door een verwijzing naar de ervaring dat het meeste SCED-onderzoek gebruik maakt van steekproefgroottes tussen de 6 en 27 deelnemers en ranges van het minimaal aantal metingen dat in de 3 fases (baseline, behandeling, follow-up) plaatsvindt. Het is onduidelijk hoe dit correspondeert met statistische power om een verschil tussen de effectiviteit van de gepersonaliseerde en standaardbehandeling te kunnen detecteren. Onduidelijk is hoe dit correspondeert met statistische power om een verschil tussen de effectiviteit van de gepersonaliseerde en standaardbehandeling te kunnen detecteren.

   Het voorliggende onderzoek lijkt echter géén SCED-onderzoek. Er lijkt sprake van een pre-post design; voor een dergelijk design kan een onderbouwing voor de sample-size veel beter uitgewerkt worden, zeker als u hypotheses wilt toetsen.

- Hopelijk hebben we met bovenstaande antwoorden duidelijk kunnen maken dat het hier om een SCED-onderzoek gaat en dat er dus geen sprake is van een vergelijking met een standaardbehandeling.
- De tekst in hoofdstuk 4.4 sample size calculation, is aangepast naar: '*As this study is a single-case experimental design with replicated measurements, a traditional sample size calculation was not performed. Experience from previous studies with SCED designs showed that the sample size varies between 6 and 27 participants (15–17). Using the results of a recent prospective cohort study, with ICU COVID-19 patients who followed an inpatient rehabilitation treatment, where the primary outcome measurement, the USER-P, improved in the first year after ICU discharge (8). In this cohort, the effect size for participation levels is medium from baseline to 12 months follow-up (>0.5) with a statistical power of >0.8. The recommended minimum number of baseline measurement in a SCED study is 5 (18). In this study, the minimum of measurement points in the baseline period is set at 7-10. With 32 measurements during the rehabilitation treatment and a minimum of 7-10 follow-up measurements. Using the calculation tool developed by Bouwmeester for SCED studies, with a medium effect size, 7-10 baseline measurements and accounting for 20% loss-to-follow-up, 20 patients will be included in this study (19)'.*

2) M.b.t. de statistiek:

- Voor de analyse van verschillen in vragenlijstscores tussen de verschillende fases wordt gebruikt gemaakt van longitudinale regressieanalyse d.m.v. linear mixed-effects models. Advies is om de resultaten voornamelijk te duiden op basis van klinische relevantie van verschillen en de mate van precisie (d.w.z. de breedte van de 95% betrouwbaarheidsintervallen) en niet teveel waarde te hechten aan p-waardes, die effectgroottes met steekproefgrootte vertroebelen.
    - We danken de METC voor dit advies. Het advies is overgenomen en de tekst in hoofdstuk 8.1 Primary study parameter(s) is aangepast naar: '*The obtained effect size will be evaluated for clinically relevant differences. As a measure of precision, we will use 95% confidence intervals*'.
- Het is niet duidelijk waar replication in het geval van dit onderzoek op slaat. Immers, iedere deelnemer ondergaat slechts eenmaal de baseline fase en eenmaal de interventiefase (in tegenstelling tot, bijvoorbeeld, een ABAB design). Het is daarom onduidelijk wat de randomisation test toevoegt boven de linear mixed-effect regressie.
    - De replicated slaat hier op de aantal SCEDs (ofwel het aantal personen dat gevolgd wordt) die we doen. We repliceren elke keer de SCED. De randomisatie test slaat op de verschillen in baseline en follow-up duur en wordt toegepast om de verschillen in dagboek scores, participatie levels en QoL tussen baseline fase, behandelfase en follow-up fase te onderzoeken. Voor elke patiënt vindt een SCED plaats en dit betreft een replicatie.
    - De tekst in hoofdstuk 8.1 Primary study parameter(s), is aangepast naar: '*In a SCED study, one outcome is repeatedly measured in the same person. In the resulting time series, hereby demonstrating*

the external validity of the effects. These replicated single-case experiments may be considered as multiple studies that can be combined using meta-analytical procedures (44). The hypothesis is that the interdisciplinary rehabilitation treatment (B) is superior to baseline (A). The null hypothesis is that there is no differential effect for any of A and any other period. The follow-up (C) is expected to be superior to A, and will not change in relation to B. A randomization test for single-case experimental designs, will be used to test whether there are statistically significant differences in participation levels and QoL between phase A versus phase B and C (baseline versus intervention plus follow-up) (44–46). With respect to Student t tests, analysis of variance F tests, or other inferential procedures from within the general linear model framework, randomization tests have the advantage of being valid for single-case experiments without making distributional assumptions of being easy to apply (46), and of being extremely versatile for even the most complex single-case design (44).

5. Eindpunten

Primaire uitkomstmaat is participatie in de samenleving (USER-P) en de gezondheidsgerelateerde kwaliteit van leven (EQ5D-5L en de bijbehorende visueel analoge schaal (VAS)). Niet duidelijk is hoe de primaire uitkomstmaten van de gepersonaliseerde interdisciplinaire revalidatie dagbehandeling in de tweede lijn vergeleken worden met de standaardzorg. Zie ook de opmerkingen onder punt 3, opzet van het onderzoek.

- Hopelijk hebben we met bovenstaande antwoorden duidelijk kunnen maken dat er geen sprake is van een standaard revalidatiegeneeskundige behandeling, maar dat er enkel gekeken wordt naar de effectiviteit van een nieuw ontworpen interdisciplinaire revalidatie behandeling in relatie tot een baseline fase, waarin geen revalidatiegeneeskundige behandeling plaatsvindt. De uitkomsten van Fase B (behandeling) worden dus vergeleken met fase A (baseline).

6. Ethische/juridische aspecten

1) Wervings- en informed consentprocedure (paragraaf 9.2 protocol): de procedure is niet voldoende duidelijk en op een aantal punten ook niet akkoord:
   - attendering op het onderzoek vindt plaats via ''consultant in rehabilitation medicine''. Is deze consultant een behandelaar? Heeft de consultant een behandelrelatie met de patiënt?
     - We danken de METC voor bovenstaande feedback. De term ‘consultant in rehabilitation medicine’ is in het protocol vervangen voor de term ‘rehabilitation physician’. De revalidatiearts ziet de patiënt de eerste keer en beoordeeld of de patiënt in aanmerking komt voor een medisch specialistische revalidatiebehandeling, in dit geval de nieuw ontworpen interdisciplinaire revalidatie behandeling voor PCS. Als een patiënt met PCS in aanmerking komt voor een medisch specialistische revalidatiebehandeling, vraagt de revalidatiearts of diegene zou willen meedoen aan het onderzoek.

De revalidatiearts is onderdeel van het behandelteam en heeft dus een behandelrelatie met de patiënt.

- De tekst in hoofdstuk 5. Treatment of subjects, is aangepast naar: '*During a first consultation with the rehabilitation physician, a decision is made if there is an indication for an interdisciplinary treatment in secondary care. If so, treatment will be personalized according to the patient's individual physical, mental and cognitive complaints and goals*'.
- Verder is de tekst in hoofdstuk 9.2 recruitment and consent, aangepast naar: '*During a first consultation with the rehabilitation physician, a decision is made if there is an indication for an interdisciplinary treatment in secondary care. If interdisciplinary rehabilitation in secondary care is indicated and the patient meeting the inclusion criteria, the rehabilitation physician will register the patient for the treatment and inform them about the aim and content of the study, both during the consultation and by providing an information letter*'.

- De consultant overhandigt de proefpersoneninformatiebrief (PIF) bij belangstelling, de onderzoeker belt de potentiële studiedeelnemer na een week om te vragen of deze belangstelling heeft om mee te doen; de potentiële studiedeelnemer heeft tussendoor altijd gelegenheid om de onderzoeker te bellen met vragen. Van belang is dat de consultant expliciet toestemming vraagt aan de potentiële studiedeelnemer om zijn/haar contactgegevens over te mogen dragen aan de onderzoeker. Verder dient geborgd te worden dat alle potentiële studiedeelnemers uitgebreide mondelinge informatie over het onderzoek krijgen van de onderzoeker en dat de bedenktijd om deelname aan het onderzoek te overwegen (minimaal 1 week) pas ingaat nadat de potentiële studiedeelnemer zowel mondelinge als schriftelijke informatie over het onderzoek heeft ontvangen.
    - We danken de METC voor de aanvullende feedback. In het protocol is duidelijker aangegeven dat de revalidatiearts vraagt of gegevens door gegeven mogen worden aan de onderzoeksassistent. De tekst in hoofdstuk 9.2 recruitment and consent, aangepast naar: '*The rehabilitation physician ask permission to give patients name and phone number to a member of the research team. Potential subjects will have 1 week to reflect before deciding whether to participate. After 1 week, they will be called by a research team member to ask whether they are willing to participate. During the telephone call, the research team member will provide the potential participant with additional information about the study and will answer any questions*'.
- bij interesse in deelname wordt aan de studiedeelnemer gevraagd om de toestemmingsverklaring te tekenen en per post te retourneren. Dat is niet akkoord. Geborgd dient te worden dat de toestemmingsverklaring in het bijzijn van de onderzoeker (i.e. op locatie) wordt getekend. Pas nadat de studiedeelnemer en de onderzoeker getekend hebben mogen de eerste

onderzoekshandelingen plaatsvinden.

  - In het protocol is aangepast dat de informed consentformulieren samen met een van de onderzoekers zal worden getekend. De tekst in hoofdstuk 9.2 recruitment and consent, aangepast naar: '*After 1 week, they will be called by a research team member to ask whether they are willing to participate. During the telephone call, the research team member will provide the potential participant with additional information about the study and will answer any questions. If the participant agrees to participate, an appointment will be made to sign the informed consent forms together with one of the researchers (Marion de Mooij (research assistant UM), Cynthia Lamper (post-doc, UM), Darcy Ummels (post-doc UM) or Carlijn Wiertz (PhD student UM))*'.

- de feasibility study kent ook een component ''focus-groep met onderzoekers''. Hiervoor is geen wervings- en informed consentprocedure uitgewerkt. Ook ontbreekt een (proefpersonen)informatiebrief voor de zorgverleners. De zorgverleners zijn ook studiedeelnemers in het voorliggende onderzoek. Van belang is dat deze groep als zodanig in alle studiedocumenten beschreven wordt (o.a. protocol, ABR, aparte informatiebrief).
  - We danken de METC voor bovenstaande opmerking. In het protocol is de werving van de zorgmedewerkers toegevoegd. Daarnaast is er ook een extra informatiebrief voor zorgverleners gemaakt (zie bijlage E2 informatiebrief zorgverleners). De tekst in hoofdstuk 9.2 recruitment and consent, is aangepast naar: '*After all included patients have completed the interdisciplinary rehabilitation treatment (phase B), a focus group lasting 1.5 hours will be organized with all disciplines of healthcare providers working with post-COVID patients within the interdisciplinary rehabilitation team. At the start of the study, all healthcare providers working with post-COVID patients within the interdisciplinary rehabilitation team (consultant in rehabilitation medicine, physiotherapists, occupational therapists, speech therapists and psychologists) will be approached personally by one of the researchers (Marion de Mooij (research assistant UM), Cynthia Lamper (post-doc, UM), Darcy Ummels (post-doc UM) or Carlijn Wiertz (PhD student UM)). To inform them about the aim and content of the study, as well verbally as by providing an information letter. The healthcare providers will have 1 week to reflect before deciding whether to participate. If the healthcare provider agrees to participate, an appointment will be made to sign the informed consent forms together with one of the researchers*'.

2) Omgaan met gegevens:
   Paragraaf 10.1 van het protocol is onvoldoende uitgewerkt.
   We danken de METC voor de opmerkingen aangaande 'omgaan met gegevens'.

   Vraag is om verder uit te werken hoe de gegevens worden verwerkt. Specifiek:

- dat en hoe er wordt gecodeerd;
    - De tekst in hoofdstuk 10.1 m.b.t. codering is nu aangevuld met: '*At enrolment, the participant will receive a participant code (unique number) and this number is written on the informed consent and will be kept locked away, separated from the rest of the research data, Data will be collected through the Your Research environment with the individualized code (unique number) such that the identity of the subjects is not available to others*'.
- wie beschikt over de sleutel van de code (Nb: de sleutel van de code mag een deelnemend centrum niet verlaten);
    - De tekst in hoofdstuk 10.1 Handling and storage of data and documents is aangevuld met: '*The researchers of the study ((Marion de Mooij (research assistant UM), Cynthia Lamper (post-doc, UM), and Darcy Ummels (post-doc UM)), the data safety monitoring board and the IGJ (Inspectie Gezondheidszorg en Jeugd) will have access to the key of the coded data. The key of the coded data will be kept at Maastricht University, department of Rehabilitation Medicine. Data will not be shared with other parties'.*
- waar de data worden bewaard en in welke vorm;
    - De tekst in hoofdstuk 10.1 Handling and storage of data and documents is aangevuld met: '*Data will be collected through the Your Research environment with the individualized code (unique number) such that the identity of the subjects is not available to others. Data is stored in a multitenant database where the study data is stored in a study specific scheme. Keys of the database are stored in KeyVault and authorization is done via Active Directory. Access is regulated by an Identity Provider (IDP), all users have an account in the identity provider, where authorization is taken care via OAuth2 and OpenID. This is also set up according to the need-to-know principle (only access to data when the person has the specific need (due to their position, role, responsibility) to see the data). Your Research uses activities for all different types of tasks (diary, questionnaire, etc.). An activity consists of questions and questions can possibly (in the case of single/multiple choice) contain answer options. These three layers (activity, questions, answer options) are stored separately. An activity contains the title of the task and options that apply (duration of completion, how long the task should remain open, indication of whether the questionnaire is mandatory etc.). This is the same as the overarching data of a task. Linked to this are the questions that apply to the specific questionnaire. For example, the symptoms to be selected from a diary, or the different questions in a questionnaire. The response options of single and multiple-choice questions are recorded separately. Therefore, a task (activity) has three levels: meta data - questions - (optional) answer options. When a task is generated for a participant there is a participant ID and activity ID, this combination also has a unique ID. The data (answers) are connected to that ID and only the GUID's of the questions and/or options are saved. After the study has been completed, Your Research will provide the entire database as a data dump to the investigators and after one*

year, they will delete the data from their server. The researchers will use the Datahub services of the UM to store the data for 15 years after the completion of the study'.

- met welk doel de gegevens worden bewaard, i.e. niet alleen voor eventuele her-analyses of nieuwe analyses die gericht zijn op het beantwoorden van de doelstellingen van het onderzoek, maar (conform de informatie in de PIF) ook - met toestemming van de studiedeelnemers – voor eventueel ander wetenschappelijk onderzoek op het gebied van langdurige klachten na corona;
  - De tekst in hoofdstuk 10.1 is aangevuld met: '*The researchers will use the Datahub services of the UM to store the data for 15 years after the completion of the study and will allow other researchers to access anonymous data for further use on the same disease. This will only be allowed if the data is requested for research into the participation levels and quality of live, and physical, mental and cognitive complaints in people with Post Covid Syndrome*'.
- wie inzage in de gegevens hebben; dit zijn in ieder geval monitors en nationale toezichthoudende autoriteiten zoals de IGJ. Graag ook toelichten of gegevens het MUMC+ (kunnen) verlaten (i.e. of er gegevens met externe partijen (kunnen) worden gedeeld) en zo ja, in welke vorm.
  - De tekst in hoofdstuk 10.1 is aangevuld met: '*The researchers of the study ((Marion de Mooij (research assistant UM), Cynthia Lamper (post-doc, UM), and Darcy Ummels (post-doc UM)), the data safety monitoring board and the IGJ (Inspectie Gezondheidszorg en Jeugd) will have access to the key of the coded data. The key of the coded data will be kept at Maastricht University, department of Rehabilitation Medicine. Data will not be shared with other parties*'. Zie ook punt 2 in vragen rondom omgaan met gegevens.

Verder: dataverwerking en -opslag vindt plaats via YourResearch. Graag nader toelichten of dit systeem state of the art is, i.e. voldoet aan AVG eisen.

- Data will be collected and processed in accordance with the General Data Protection Regulation (EU) 2016/679). Please see: Compliance with national requirements on Data Protection section for more information. Your Research protects its systems by using the most recent standards and is ISO 27001 and NEN 7510 certified (standard of information security). If Your Research has a data breach, the following steps will occur, the processor refers to Your Research and the Controller refers to the research institute that has an agreement with Your Research:

1. If the Processor becomes aware of an incident that may have a (material) impact on the security of Personal Data, it will i) notify the Controller without unreasonable delay and ii) take all reasonable measures to prevent or limit (further) violation of the GDPR.
2. Insofar as is reasonable, the Processor will cooperate with the Controller and support the Controller in the performance of its legal obligations in respect of the identified incident.
3. Insofar as is reasonable, the Controller will support the Processor in its obligation to report the personal data breach to the Dutch Data Protection Authority ("Dutch DPA") and/or the data subject, as referred to in Article 33, paragraph 3 and Article 34, paragraph 1 of the GDPR. The Processor is never obliged to independently notify a personal data breach to the Dutch DPA and/or the data subjects.
4. The Processor is never liable for correct and/or timely performance of the obligation to

notify to which the Controller is subject as referred to in Articles 33 and 34 of the GDPR.

5. The trial team will then inform the participants of the study if there is a high chance of risk to the rights and freedom of the participants in the study due to the data breach.
6. If Datahub has a security breach, they will refer to the sections of the GDPR that concern data breaches. They will follow all the required regulations in that case.
   - In hoofdstuk 10.1 is dit weergegeven als: '*Data will be collected and processed in accordance with the General Data Protection Regulation (EU) 2016/679). Your Research protects its systems by using the most recent standards and is ISO 27001 and NEN 7510 certified (standard of information security)*'.

7. Publicatieparagraaf
   Graag toevoegen dat publicatie zal geschieden conform het CCMO-statement ''Publicatiebeleid''. Verder graag verwijzen naar het (nog aan te leveren) onderzoekscontract voor de publicatievoorwaarden.
   - De tekst in hoofstuk 10.6 Public disclosure and publication policy is aangepast naar: '*Publication Policy is in agreement with the CCMO publication statement. The results of the study will be published in scientific articles in peer-reviewed scientific journals, regardless the positive or negative results (see Appendix K3 Consortiumovereenkomst PINCOR)*'.

Publicatie zal geschieden volgens de consortiumovereenkomst die door ZonMw gefinancierde projecten is gesloten tussen Universiteit Maastricht, Stichting Radboud Universitair Centrum en de Stichting Adelante Zorg (zie bijlage K3 Consortiumovereenkomst PINCOR). Op woensdag 31-05 is telefonisch contact geweest vanuit de PINCOR onderzoeksgroep met ambtelijk secretaris mevr. dr. E.C.H. van den Ham. Haar advies was om toch de standaard van het CCMO aan te houden als onderzoekscontract tussen Adelante en UM specifiek m.b.t de aspecten ter beoordeling: het voortijdig beëindigen van het wetenschappelijk onderzoek of de overeenkomst en de openbaring van resultaten van het wetenschappelijk onderzoek. Na overleg binnen de projectgroep zou het nieuwe op te stellen contract volgens CCMO template interfereren met de eerder opgestelde consortiumovereenkomst voor het gehele PINCOR project. Mede op advies van de ambtelijk secretaris hebben we contact opgenomen met de afdeling juridische zaken van de UM (mevr. Suzanne ten Hoeve) en de vraag of het consortiumovereenkomst ook afdoende is t.a.v. het voortijdig beëindigen en de publicatie(s). Dit wordt nu opgepakt, maar door drukte bij de juridische afdeling volgt reactie op korte termijn en zullen wij zo spoedig mogelijk aanvullende informatie verstrekken.

<u>Met betrekking tot de proefpersoneninformatiebrief (PIF) en de toestemmingsverklaring:</u>

1. Algemeen:
   De PIF dient in lijn te zijn met de opzet / het design van het onderzoek. Opzet / design is niet duidelijk (zie alle opmerkingen hieromtrent bij de belangrijkste vragen en opmerkingen bij het protocol); dat maakt dat de commissie de PIF niet goed kan beoordelen. Verder: het template voor PIF en de toestemmingsverklaring is niet overal gevolgd. Voor het opstellen van de revisie van de PIF graag de meest recente versie van het Model proefpersoneninformatie gebruiken (zie voor het Model en meer informatie

over het gebruik van dit Model: website CCMO). De commissie zal de PIF en de toestemmingsverklaring t.z.t. opnieuw integraal beoordelen.

- We danken de METC voor de aanvullende feedback. We hebben de PIF aangepast, waarbij het design van de studie beter is omschreven en het template voor de PIF aangepast, conform de template (E1/E2. Model proefpersoneninformatie voor proefpersonen van 16 jaar en ouder (volwassenen), via de site van het CCMO). Zie voor alle aanpassingen de track changes in de PIF in combinatie met onderstaande antwoorden.

2. Punten die in ieder geval aandacht behoeven bij het opstellen van de revisie van de PIF en toestemmingsverklaring:
   1) M.b.t. paragraaf 2, doel van het onderzoek: hoe wordt “goed” beoordeeld? Wat is ‘’goed’’?
      a. De tekst in paragraaf 2 is aangepast naar: ‘*In dit onderzoek willen we beoordelen wat voor een effect een revalidatie dagbehandeling heeft op het weer kunnen uitvoeren van dagelijkse activiteiten, kwaliteit van leven en verschillende klachten die kunnen voorkomen bij het post-COVID-syndroom. Daarnaast willen we onderzoeken of de behandeling toe te passen is bij mensen die langdurig klachten hebben na COVID-19*’.
   2) M.b.t. paragraaf 3: de titel van deze paragraaf graag wijzigen in ‘’Achtergrond van het onderzoek’’. Verder ook hier graag meer aandacht voor het wetenschappelijk belang van het voorliggende onderzoek (zie ook de belangrijkste vragen en opmerkingen m.b.t. het protocol).
      a. De tekst in paragraaf 3 is aangepast, zie de track changes.
   3) M.b.t. paragraaf 4, wat houdt deelname in: er lijken ook gegevens uit het medisch dossier van de studiedeelnemer opgevraagd te worden (bijv. gegevens van vragenlijsten die reeds zijn afgenomen in het kader van de reguliere zorg; zie paragraaf 10 van de PIF). Graag in paragraaf 4 kort beschrijven dat u gegevens uit het medisch dossier wilt opvragen uit het medisch dossier, om welke gegevens het hier dan gaat en dat u hiervoor toestemming vraagt aan de studiedeelnemer (Nb: in de toestemmingsverklaring een passage hieromtrent toevoegen).
      a. De tekst in paragraaf 4 is aangepast, zie de track changes.
      b. De toestemmingsverklaring is aangepast, zie de track changes.
   4) Paragraaf 8, einde van het onderzoek: bij de laatste punt van de opsomming van situaties waarin het onderzoek stopt: Adelante zorggroep graag vervangen door Maastricht University (een deelnemend centrum kan niet besluiten dat het onderzoek moet stoppen, dat kan alleen de verrichter van het onderzoek, de overheid of de METC die het onderzoek heeft beoordeeld);.
      a. De tekst in paragraaf 8 is aangepast, zie track changes.
   5) Paragraaf 10: graag volledig herzien en voor het opstellen van deze paragraaf graag gebruik maken van de standaardteksten uit het Model proefpersoneninformatie (Nb: ook graag de titel van deze paragraaf graag aanpassen).
      a. De tekst in paragraaf 10 is aangepast, zie track changes.
   6) ABR F3 vermeldt dat de huisarts geïnformeerd wordt over de deelname van de patiënt aan het onderzoek. E.e.a. is niet opgenomen in de voorliggende PIF en toestemmingsverklaring.
      a. De tekst in de PIF en de toestemmingsverklaring is aangepast. Hierbij wordt nu benoemd dat we de huisarts en behandelend revalidatiearts informeren over deelname aan de studie.

7) Bijlage C, informatie over de verzekering, is niet ingevuld. Maastricht University is de verrichter van het onderzoek en derhalve ook verantwoordelijk voor het afsluiten van de WMO-proefpersonenverzekering. Graag de gegevens van de WMO-proefpersonenverzekering van Maastricht University opnemen (zie website METC azM/UM: model 2 verzekeringsverklaring).
    a. In bijlage C is aangepast dat Maastricht University de verrichter van het onderzoek is en daarmee ook de verzekering heeft afgesloten. De gegevens van de WMO-proefpersonenverzekering van Maastricht University zijn aangevuld.

3. Zie ook eerder: de feasibility study omvat ook een component voor hulpverleners. Graag een PIF en toestemmingsverklaring voor de hulpverleners aanleveren.
    a. Zoals eerder ook al is beschreven is een PIF en toestemmingsverklaring voor de hulpverleners toegevoegd.

Onderzoekscontract
Een onderzoekscontract tussen Maastricht University (verrichter) en Adelante ontbreekt bij de door u aangeleverde documenten. U wordt gevraagd dit contract aan de commissie te doen toekomen. Voor een template en meer informatie over het indienen van het onderzoekscontract verwijzen wij u graag naar de website van de CCMO (https://www.ccmo.nl/onderzoekers/standaardonderzoeksdossier/k-overige-documenten/k3-onderzoekscontracten).

- We hebben een nieuwe onderzoeksverklaring van Adelante aangeleverd. De aansprakelijkheid van degenen die het onderzoek uitvoeren voor schade door dood of letsel van de proefpersoon, valt onder de dekking van de aansprakelijkheidsverzekering van Stichting Adelante Groep, die is afgesloten bij Centramed (zie bijlage, I2 Onderzoeksverklaring Adelante – PINCOR v2)

Na ontvangst van uw reactie en een exemplaar van de aangepaste documenten zal de commissie zo spoedig mogelijk uw reactie bespreken. Op http://toetsingonline.ccmo.nl kunt u de stand van zaken van uw beoordeling volgen. De commissie verwacht dat u binnen drie maanden na dagtekening reageert. Na deze termijn zal zij uw voorstel seponeren, tenzij u een met redenen omkleed verzoek indient om de behandeling aan te houden.

Met vriendelijke groet, namens de METC azM/UM,

mevr. dr. E.C.H. van den Ham,
ambtelijk secretaris

prof. dr. H.C. Schouten,
vicevoorzitter

Kopie: Prof. dr. J.A. Verbunt, Department of Rehabilitation Medicine, Maastricht University

Bijlage 1: Administratieve aspecten

Bijlage 1: Administratieve aspecten

<u>Met betrekking tot het onderzoeksprotocol</u>:

1. In de tabel op pagina 3 wordt Adelante Zorggroep vermeld bij ‘’Laboratory sites”, maar in het voorliggende onderzoek lijkt geen lichaamsmateriaal verzameld te worden. Graag uw nadere toelichting.
    a. Er wordt geen lichaamsmateriaal verzameld. Adelante Zorggroep is verwijderd bij ‘’Laboratory sites”.
2. M.b.t. paragraaf 6.7: voor redenen voor voortijdige beëindiging graag verwijzen naar het nog aan te leveren onderzoekscontract.
    a. Zoals eerder reeds beschreven zijn wij nog in afwachting van de beoordeling van de juridische zaken van de UM inzake de consortiumovereenkomst t.a.v. de te beoordelen aspecten: het voortijdig beëindigen van het wetenschappelijk onderzoek of de overeenkomst en de openbaring van resultaten van het wetenschappelijk onderzoek. wij hopen hier op korte termijn respons van te krijgen en dan ook zo spoedig mogelijke aanvullende informatie te kunnen verstrekken.
3. M.b.t. paragraaf 7.2.1: de formulering ‘’related to the experimental intervention’’ suggereert dat de tweedelijns interdisciplinaire revalidatiebehandeling afwijkt van de standaardzorg; is dat correct?
    a. Momenteel is er nog geen standaard zorg binnen de revalidatiegeneeskunde voor de behandeling van post-COVID syndroom. Fase A is de baseline fase, waarbij geen behandeling wordt aangeboden. In fase B start de nieuw ontworpen interdisciplinaire revalidatie behandeling.
4. M.b.t. paragraaf 7.3: de laatste zin graag vervangen door ‘’SAEs need to be reported till end of study within the Netherlands, as defined in the protocol‘’.
    a. De tekst is aangepast.
5. M.b.t. paragraaf 9.1: wat wordt bedoeld met ‘’logical guidelines’’?
    a. We hebben logical guidelines verwijderd.
6. M.b.t. paragraaf 10.2:
   (1) in UMC ’s is monitoring verplicht voor alle onderzoek dat onder de reikwijdte van de WMO valt. Graag beschrijven wie de monitoring verzorgt, wat gemonitord wordt, frequentie van de monitoring etc. Voor details kan verwezen worden naar het monitoring plan. Gebruikelijk is dat monitoring van studies waarvan Maastricht University de verrichter is, wordt verzorgd door het CTCM;
   (2) informatie over het omgaan met gegevens hier graag verwijderen; deze informatie hoort thuis in paragraaf 10.1. Aandachtspunten: a) m.b.t. de passage ‘’All researchers can export .. YourResearch’’ > het gaat hier om gecodeerde data en niet om geanonimiseerde data; b) m.b.t. de passage “Marion … non-anonymised data’’ > om welke niet-geanonimiseerde data gaat het en waar worden deze data bewaard?
    - (1) De monitoring zal verzorgd worden door CTCM. De tekst in hoofdstuk 10.2 Monitoring and Quality Assurance is aangevuld met: ‘*The risks attached to participating in this study are negligible and, in any case, no greater than no greater compared with other interdisciplinary rehabilitation treatment (e.g. for chronic pain or stroke). In total, 20 participants need to be included in this SCED*

study in only one centre (Adelante). The monitoring will be provided by the CTCM and conducted by an independent researcher from Adelante. Based on these facts, this study needs minimal monitoring due to the negligible risk according the NFU guidelines "Kwaliteitsborging mensgebonden onderzoek 2.0". Therefore, on-site visitation is only needed once in the coordinating centre, and one remote visitation in the participation centre. Informed consent forms and source data verification will also take place for 10% of all subjects (n = 2 of 10 participants). 5% of the SAEs will be monitored. The monitored data will comprise: age, sex, questionnaires and dairies'.

- (2) De informatie is verwijderd uit hoofdstuk 10.2 en de tekst in hoofdstuk 10.1 is aangepast, zie vorige opmerkingen.

7. M.b.t. paragraaf 10.3: de tweede alinea graag in het geheel verwijderen.
    a. De tekst is verwijderd.

<u>Met betrekking tot het ABR-formulier</u>:

1. B6: Maastricht University is de verrichter van het voorliggende onderzoek. Conform B5 is mw. Lamper werkzaam bij Maastricht University. Vraag B6 derhalve graag met ''ja'' beantwoorden.
    a. Aangepast zoals voorgesteld
2. C23: de startdatum aanpassen aan de datum van beoogde inclusie van de eerste proefpersoon.
    a. De start- en einddatum zijn aangepast
3. D4/D5: graag aanpassen conform de opmerkingen bij het protocol.
    a. Aangepast conform protocol
4. D10: graag verifiëren of e.e.a. correct is ingevuld.
    a. Dit is geverifieerd en dat klopt. De revalidatiearts informeert de patiënt enkel over het onderzoek en vraagt toestemming of de patiënt benadert mag worden vanuit het onderzoeksteam. Het onderzoeksteam vraagt de patiënt voor deelname.
5. E4: zie eerder: is de tweedelijns interdisciplinaire revalidatiebehandeling standaardzorg? Of is er sprake van een experimentele interventie?
    a. Momenteel is er nog geen standaard zorg binnen de revalidatiegeneeskunde voor de behandeling van post-COVID syndroom. Fase A is de baseline fase, waarbij geen behandeling wordt aangeboden. In fase B start de nieuw ontworpen interdisciplinaire revalidatie behandeling.
6. E9: zie eerder: de opmerking ''de inhoud van de interventie is *gebaseerd* op de reguliere zorg'' impliceert dat er sprake is van een experimentele interventie (dus niet reguliere zorg).
    a. Momenteel is er nog geen standaard zorg binnen de revalidatiegeneeskunde voor de behandeling van post-COVID syndroom. Fase A is de baseline fase, waarbij geen behandeling wordt aangeboden. In fase B start de nieuw ontworpen interdisciplinaire revalidatie behandeling.
7. F1: graag aanpassen conform de opmerkingen bij het protocol.
    a. Aangepast conform protocol
8. F2: graag in overeenstemming brengen met het protocol (*minimaa*l 1 week).
    a. Aangepast zoals voorgesteld
9. F4a: graag vermelden *hoe* de code is opgebouwd. Nb: de passage ''an automatic made participant code by YourResearch so that all data will be recorded anonymously'' is niet

correct. Gecodeerde data zijn geen anonieme data.

   a. De opbouw van de code is nu aangepast in het ABR formulier.

10. F4b: de vraag graag beantwoorden: wie beschikt over de sleutel van de code?
    a. Aangepast in het ABR formulier
11. F4c: zie ook de opmerking bij F4a: gecodeerde data zijn niet anoniem! Graag aanpassen. Graag toevoegen dat naast de leden van het onderzoeksteam ook monitors en toezichthoudende autoriteiten toegang tot de brondocumenten en andere tot de persoon herleidbare gegevens kunnen hebben.
    a. Aangepast in het ABR formulier
12. G3: de vergoeding die u ontvangt voor de uitvoering van het onderzoek is bijna 500.000 euro, dat lijkt buitenproportioneel veel voor een interview/vragenlijst studie met 20 deelnemers; e.e.a. impliceert dat de behandeling geen standaardzorg is.
    a. Aangepast in het ABR formulier, de arts/onderzoeker ontvangt geen vergoeding
13. Samenvatting: graag aanpassen conform de opmerkingen bij het protocol.
    a. Aangepast conform protocol.

M.b.t. de onderzoeksverklaring

Graag een nieuwe onderzoeksverklaring van Adelante aanleveren. Van belang is dat op de verklaring vermeld wordt dat de aansprakelijkheid van degenen die het onderzoek uitvoeren voor schade door dood of letsel van de proefpersoon, valt onder de dekking van de aansprakelijkheidsverzekering van Adelante. Tevens dient vermeld te worden waar Adelante de aansprakelijkheidsverzekering heeft afgesloten.

- We hebben een nieuwe onderzoeksverklaring van Adelante aangeleverd. De aansprakelijkheid van degenen die het onderzoek uitvoeren voor schade door dood of letsel van de proefpersoon, valt onder de dekking van de aansprakelijkheidsverzekering van Stichting Adelante Groep, die is afgesloten bij Centramed (zie bijlage, I2 Onderzoeksverklaring Adelante – PINCOR v2).
