# Supplementary material for: Effectiveness of a Person-Centered Interdisciplinary Rehabilitation Treatment of Post–COVID-19 Condition: Protocol for a Single-Case Experimental Design Study
Source: JMIR Res Protoc. 2024 Oct 11;13:e63951. doi: 10.2196/63951 (PMC11512124; doi:10.2196/63951)
Supplement: Multimedia Appendix 3 [file resprot_v13i1e63951_app3.pdf]

Dr. C. Lamper
Department of Rehabilitation Medicine
Maastricht University

ons kenmerk 23.0013288
doorkiesnummer 043-3876009
datum 27/06/2023

Betreft: Reactie n.a.v. bespreking in METC azM/UM – NL83848.068.23 / METC23-008
Titel: Effectiveness of an Interdisciplinary rehabilitation treatment for patients with post-COVID syndrome

Geachte dr. Lamper,

De medisch-ethische toetsingscommissie (METC) azM/UM heeft bovengenoemd onderzoeksvoorstel voor de tweede keer besproken in haar vergadering van 14-06-2023. De eerste bespreking vond plaats op 08-03-2023.

De commissie heeft nog enkele vragen en opmerkingen met betrekking tot het protocol en de proefpersoneninformatie. Verder heeft zij nog een aantal vragen van administratieve aard; deze vragen treft u aan in de bijlage.

Voor een vlotte afhandeling wordt u gevraagd in uw reactiebrief de vragen van de commissie over te nemen en te laten volgen door uw antwoord met waar nodig verwijzingen naar aanpassingen in de documenten. Er kunnen alleen documenten in behandeling genomen worden waarin wijzigingen door middel van onderstreping zijn gemarkeerd (m.u.v. het ABR-formulier). Ieder document dient voorzien te zijn van een paginanummer en een versiedatum.

Belangrijkste vragen en opmerkingen

Met betrekking tot het onderzoeksprotocol:

1. Opzet
   - De voorliggende studie beoogt het effect van een gepersonaliseerde interventie te meten. Zowel de WHO- als de NICE-richtlijn adviseren een interdisciplinair revalidatietraject bij PCS patiënten die ernstig beperkt zijn (protocol referenties 1 en 3). Potentiele deelnemers voor de studie worden geput uit de populatie die verwezen is voor een interdisciplinaire revalidatiebehandeling in de 2e lijn. Wordt onafhankelijk van het wel of niet meedoen aan de voorliggende studie dezelfde gepersonaliseerde interventie/behandeling aangeboden aan iemand die verwezen is voor een interdisciplinaire revalidatiebehandeling in de 2e lijn?
     - We danken de METC voor haar opmerkingen. Het klopt dat onafhankelijk van het wel of niet meedoen aan de studie een patiënt die verwezen wordt voor een interdisciplinaire revalidatie behandeling binnen de tweede lijn, dezelfde gepersonaliseerde behandeling krijgt aangeboden. Binnen de

revalidatiegeneeskunde wordt al bij veel verschillende diagnosegroepen gebruik gemaakt van een gepersonaliseerde interdisciplinaire revalidatie behandeling. Echter is er momenteel nog geen standaard zorg voor PCS binnen de revalidatiegeneeskunde. Door deze studie willen de dan ook de effectiviteit van een gepersonaliseerde interdisciplinaire revalidatie behandeling aantonen bij PCS.

- Als men deelneemt aan de studie, wordt de start van de behandeling met 3-5 weken uitgesteld (fase A 2-4 weken wachttijd, plus een week bedenktijd om deelname aan de studie te overwegen). In de PIF dient helder en duidelijk beschreven te worden dat de behandeling bij deelname aan het onderzoek 3-5 weken later start dan bij niet deelnemen aan de studie.
    - We danken de METC voor bovenstaande opmerking. Het klopt dat de wachttijd tot de start van de definitieve revalidatie behandeling ongeveer 3-5 weken duurt. Echter is het zo dat er momenteel binnen de medisch specialistische revalidatie zorg (Adelante, locatie MUMC+) ook een wachttijd bestaat van 4-6 weken. Dit houdt in dat indien een patiënt gezien is door revalidatiearts en geïndiceerd wordt voor een interdisciplinaire revalidatiebehandeling het ook gemiddeld 4-6 weken duurt voor deze behandeling start. Hiermee zit er dus nauwelijks verschil in wachttijd tussen start van de behandeling afhankelijk of iemand wel of niet deelneemt aan de studie. In de PIF voor patiënten is de volgende tekst toegevoegd aan hoofdstuk 4, hoe verloopt het onderzoek: '*De wachttijd van het onderzoek is vergelijkbaar met de duur van de reguliere wachtlijst voor het kunnen starten van een poliklinische revalidatiebehandeling. De definitieve revalidatiebehandeling start dan ook niet of nauwelijks later dan als u niet mee zou willen doen met het onderzoek*'.

2. Statistiek
Paragraaf 8.1 van het protocol (primary study parameters) is op een aantal punten niet helemaal duidelijk. Wat wordt bedoeld met "In the resulting time series hereby demonstrating hereby demonstrating the external validity of the effects"? En wat wordt bedoeld met "…there is no differential effect for any of A and any other period"?

- We danken de METC voor aanvullende vragen rondom de statistische analyse. Een beperking van de SCED is de generaliseerbaarheid van de bevindingen. We onderzoeken de effecten van behandeling immers op individueel niveau. Om de generaliseerbaarheid te vergroten repliceren we de onderzoeksopzet in n=20 individuen. De 'within-subject data' van deze replicaties worden daarbij gecombineerd middels multilevel meta-analyse technieken. De tekst in hoofdstuk 8.1 Primary study parameter(s) is aangepast naar:' *In a SCED study, one outcome is repeatedly measured in the same person. These replicated single-case experiments may be considered as multiple studies that can be combined using meta-analytical procedure, which will increase the generalizability of the study findings (44)*'.
- De nulhypothese is dat de interdisciplinaire revalidatie behandeling geen effect laat zien op de primaire en secundaire uitkomstmaten. Onze hypothese is echter dat de interdisciplinaire revalidatie behandeling een positief effect wat betreft kwaliteit van leven en participatie. Wij hopen dan ook uiteindelijk de nulhypothese te kunnen verwerpen. De tekst in het protocol is aangepast naar: '*The null hypothesis is that there is no differential effect of the interdisciplinary rehabilitation treatment. The hypothesis*

of the study is that the interdisciplinary rehabilitation treatment (B) is superior to baseline (A) and that the follow-up (C) is expected to be superior to A, and will not change in relation to B'.

3. Ethische/juridische aspecten
   1) Wervings- en informed consentprocedure:
      De procedure voor de patiënten vereist nog aanpassing:
      - Geborgd dient te worden dat alle potentiële deelnemers uitgebreide mondelinge informatie over het onderzoek ontvangen van de onderzoeker en dat de bedenktijd om deelname aan het onderzoek te overwegen ingaat nadat de potentiële deelnemer deze uitgebreide mondelinge informatie over het onderzoek heeft ontvangen van de onderzoeker. Dus: eerst uitgebreide mondelinge informatie door de onderzoeker, vervolgens 7 dagen bedenktijd voor de potentiële deelnemer om deelname aan het onderzoek te overwegen en pas na het verstrijken van de bedenktijd een telefoontje van de onderzoeker met de vraag of de potentiële deelnemer nog vragen heeft en of hij/zij wil deelnemen.
      - Niet duidelijk is waar de toestemmingsverklaring wordt getekend.
        - De eerste uitgebreide mondelinge informatie over de studie wordt gegeven door de revalidatiearts. De revalidatiearts is volledig op de hoogte van doel, inhoudt en organisatie van de studie. De revalidatieartsen die PCS-patiënten zien op locatie MUMC+ zijn beide betrokken binnen het onderzoeksteam. De revalidatiearts moet hierdoor ook eventuele vragen van de patiënt direct kunnen beantwoorden. Zeven dagen na de mondelinge uitleg van de revalidatiearts wordt de patiënt gebeld door een andere medewerker van het onderzoeksteam, waarbij aanvullende vragen worden beantwoord en gevraagd wordt of iemand definitief wel/niet wil deelnemen. Indien iemand wilt deelnemen aan het onderzoek wordt een gezamenlijke afspraak gepland voor het tekenen van de toestemmingsverklaring. De toestemmingsverklaring zal worden getekend op de revalidatieafdeling van het MUMC+. De volgende tekst is toegevoegd aan hoofdstuk 9.2: '*The informed consent form will be signed at location MUMC+, rehabilitation department*'.

      Protocol paragraaf 9.2 en ABR-formulier F1 graag aanpassen en verduidelijken op bovengenoemde punten.
        - Zowel de tekst in het protocol, hoofdstuk 9.2 als het ABR-formulier zijn aangepast.
   2) M.b.t. het omgaan met gegevens (paragraaf 10.1 protocol):
      - Waarom hebben 3 mensen toegang tot de sleutel van de code nodig? Het aantal mensen dat toegang heeft tot de sleutel van de code dient tot een minimum beperkt te zijn. Graag uw nadere toelichting.
        - We danken de METC over de opmerkingen aangaande omgaan met gegevens. Op dit moment hebben 3 mensen toegang tot de sleutel. Vanwege een zwangerschapsverlof en vervanging daarvan hebben beide post-doc medewerkers toegang tot de sleutel. In de praktijk zal echter maar een van de post-doc medewerkers toegang hebben tot de sleutel. Voor de volledigheid zijn wel beide medewerkers in het protocol opgenomen. De tekst inzake inzage in

gegevens is aangepast naar: '*The researchers of the study, research assistant UM (Marion de Mooij) and post-doc, UM (Cynthia Lamper or Darcy Ummels), will have access to the key of the coded data*'.

- Inzage in gegevens en code loopt door elkaar: een beperkt aantal onderzoekers heeft toegang tot de sleutel van de code. Leden van de DSMB, monitors en toezichthoudende autoriteiten kunnen voor controledoeleinden toegang hebben tot de gegevens (i.e. zowel de gecodeerde gegevens als tot de proefpersonen herleidbare gegevens). E.e.a. graag als zodanig vermelden in het protocol. Verder: in de opsomming van personen die ter controle toegang kunnen hebben tot de gegevens nog de leden van de veiligheidscommissie verwijderen; er is immers geen veiligheidscommissie ingesteld voor het voorliggende onderzoek.
  - De leden van de DSMB zij verwijderd uit het protocol, aangezien die geen toegang zullen hebben tot de data. De monitoring vanuit de CTCM is toegevoegd. De tekst in hoofdstuk 10.1 is aangepast naar: '*The monitoring of CTCM (Clinical Trial Center Maastricht) and the IGJ (Inspectie Gezondheidszorg en Jeugd) can get access to all data for monitoring purpose*'.
- Hoe komt u aan de medische gegevens? Er dient expliciet toestemming aan de proefpersoon gevraagd te worden om medische gegevens op te mogen vragen bij de behandelaar. In de toestemmingsverklaring graag de volgende passage toevoegen: 'Ik geef toestemming om de onderzoeker toestemming om informatie op te vragen bij mijn huisarts/specialist(en) die mij behandelt/over […].
  - In de toestemmingsverklaring is de volgende passage toegevoegd: '*Ik geef de onderzoeker toestemming om medische informatie op te vragen bij mijn revalidatiearts die mij behandelt voor het post-COVID-syndroom*'.

4. Publicatie
   In paragraaf 10.6 in de tweede zin de verwijzing naar de consortiumovereenkomst PINCOR graag verwijderen. Graag de volgende tekst toevoegen: ''Voor publicatievoorwaarden: zie onderzoekscontract".
   - De verwijzing naar de consortiumovereenkomst is verwijderd. De volgende tekst is aan hoofdstuk 10.6 toegevoegd: '*For publication conditions: see research contract, appendix K3; Clinical study site agreement PINCOR*'.

<u>Met betrekking tot de PIF en toestemmingsverklaring voor de patiënten:</u>

1. In paragraaf 6, bij de nadelen vermelden dat deelname aan de studie leidt tot een vertraging van de start van de behandeling van 3 tot 5 weken (Zie ook de opmerkingen bij punt 1 bij de belangrijkste vragen en opmerkingen m.b.t. het protocol).
   - We danken de METC over de opmerkingen aangaande de wachtduur. Zie ook het antwoord bij punt 1, opzet van de studie. De wachttijd van de studie is vergelijkbaar met de reguliere wachttijd (bij wachtlijsten) voor het starten van een interdisciplinaire revalidatiebehandeling. Dit is ook zo verduidelijkt in de PIF voor patiënten, die aanpassingen hoofdstuk 4, hoe verloopt het onderzoek.
2. M.b.t. paragraaf 4, hoe verloopt het onderzoek:
   - In uw reactiebrief en in het protocol is het misverstand van vergelijking met 'care as usual' opgehelderd. Er wordt in het voorliggende onderzoek bij elk individu vergeleken met baseline zonder behandeling ('wachttijd' genoemd in de PIF). In de PIF wordt

echter niet uitgelegd wat de bedoeling van de wachttijd is: waarom is de wachttijd(fase) in het voorliggende onderzoek belangrijk? Dit graag uitleggen in de PIF. Iets soortgelijks geldt voor de follow-up periode: in de PIF staat dat er dan geen behandeling meer plaatsvindt, maar het onderzoek loopt nog door in deze periode. Van belang is dat voor de deelnemers duidelijk is wat er in de follow-up periode nog gebeurt: op het einde van de follow-up periode (i.e. 3 maanden na het einde van de revalidatiebehandeling) wordt er nog een vragenlijst afgenomen. E.e.a. graag verduidelijken in de PIF.

- De opzet van de studie is in de PIF uitgebreider omschreven. Aangaande de wachttijd is de volgende tekst is toegevoegd aan hoofdstuk 4, hoe verloopt het onderzoek: '*Bij start van de wachttijd vult u eenmalig een uitgebreide vragenlijst in en 7-10 keer een kort dagboekje. De wachttijd wordt gebruikt om het effect van de behandeling te kunnen vergelijken met een periode dat er geen behandeling plaatsvindt'*. Wat betreft de follow-up fase is de volgende tekst toegevoegd aan de PIF: '*Wel wordt u gevraagd om 3 maanden na afronding van de poliklinische revalidatie dagbehandeling nog een uitgebreide vragenlijst en 7-10 dagboekjes in te vullen. Dit zal worden gebruikt om te beoordelen of het effect van de behandeling ook aanhoudt na afronding van de behandeling'*.

- M.b.t. "Medische gegevens'': gegevens als naam, geslacht en e-mailadres worden niet uit het medisch dossier gehaald. Graag verwijderen. M.b.t. het e-mailadres: wordt dit alleen door de onderzoekers gebruikt of ook door derden. Bijv. om vragenlijsten te kunnen versturen? In het laatste geval dient de deelnemer hierover geïnformeerd te worden in paragraaf 10 van de PIF (Zie ook de toelichtingen in de meeste recente versie van het Model proefpersoneninformatie op de website van de CCMO). Verder: hier graag vermelden dat u de genoemde gegevens graag <u>*wilt*</u> opvragen uit het medische dossier en dat u hiervoor toestemming vraagt aan de deelnemer.
  - Met betrekking tot het verzamelen van medische gegevens zijn gegevens als naam, adres, geslacht en e-mailadres verwijderd. In hoofdstuk 4 hoe verloopt het onderzoek de volgende zin aangepast: '*Doet u mee aan het onderzoek? Dan willen we graag we de volgende gegevens uit uw medisch dossier opvragen. Wij vragen hiervoor uw toestemming*'.
  - Wat betreft het gebruik van het e-mailadres is de volgende zin toegevoegd aan paragraaf 10: '*Gegevens kunnen worden gebruikt door de opdrachtgever (de universiteit van Maastricht) en bedrijven die de opdrachtgever helpen bij het uitvoeren van de studie (uw e-mailadres wordt gebruikt door YourResearch voor het verzenden van de vragenlijsten en dagboekjes)*'.

3. M.b.t. paragraaf 10, wat doen we met uw gegevens:
   - De eerste zin graag wijzigen in: "Dan geeft u ook toestemming om uw gegevens te verzamelen, te gebruiken en te bewaren".
     - De eerste zin is aangepast naar: '*Doet u mee aan het onderzoek? Dan geeft u ook toestemming om uw gegevens te verzamelen, te gebruiken en te bewaren'*.
   - In de sub-paragraaf "Waarom verzamelen, gebruiken en bewaren we uw gegevens?'' graag de tekst 'Ook hebben we deze gegevens nodig om de onderzochte poliklinische revalidatie behandeling op de markt te kunnen brengen" verwijderen.
     - De zin '*Ook hebben we deze gegevens nodig om de onderzochte poliklinische revalidatie behandeling op de markt te kunnen brengen'* is verwijderd uit paragraaf 10.
   - In de sub-paragraaf "Wie kunnen uw gegevens zien?'' in de opsomming van personen die ter controle bij de gegevens kunnen komen de passage ''Enkele leden van het

onderzoeksteam (dit zijn; Marion de Mooij, Cynthia Lamper en Darcy Ummels)'' verwijderen.

  - De toevoeging van namen welke leden van het onderzoeksteam '*dit zijn; Marion de Mooij, Cynthia Lamper en Darcy Ummels*' is verwijderd uit paragraaf 10.

- In de sub-paragraaf "Hoe lang bewaren we uw gegevens?'': ''geanonimiseerde'' verwijderen.
  - De zin in paragraaf 10, hoelang bewaren we uw gegevens is aangepast naar: '*We bewaren uw gegevens 15 jaar, binnen de Universiteit van Maastricht'*.
- In de sub-paragraaf ""Wilt u meer weten over uw privacy?'': wijzigen dat Maastricht University en Adelante verantwoordelijk zijn voor de verwekring van de gegevens (en niet de FG van de UM en de klachtenfunctionaris). Verder de passage '' Bij vragen of klachten over … klacht in bij de Autoriteit Persoonsgegevens'' graag vervangen door ''Als u klachten heeft over de verwerking van uw persoonsgegevens, raden we u aan om deze eerst te bespreken met het onderzoeksteam. U kunt ook naar de Functionaris Gegevensbescherming van de instelling gaan. Of u dient een klacht in bij de Autoriteit Persoonsgegevens''.
  - De tekst in paragraaf 10, wilt u meer weten over uw privacy is aangepast naar: '*Meer informatie over uw rechten bij de verwerking van persoonsgegevens? Kijk dan op www.autoriteitpersoonsgegevens.nl. Heeft u vragen over uw rechten? Of heeft u een klacht over de verwerking van uw persoonsgegevens? Neem dan contact op met degene die verantwoordelijk is voor de verwerking van uw persoonsgegevens. Voor uw onderzoek is dat Maastricht Universiteit en Adelante. Als u klachten heeft over de verwerking van uw persoonsgegevens, raden we u aan om deze eerst te bespreken met het onderzoeksteam (zie bijlage A). U kunt ook naar de Functionaris Gegevensbescherming van [Universiteit Maastricht of Adelante gaan (zie bijlage A). Of u dient een klacht in bij de Autoriteit Persoonsgegevens'*.
- In de sub-paragraaf ''Waar vindt u meer informatie over het onderzoek?' Nog toevoegen waar de deelnemer het onderzoek kan vinden (i.e. ''U vindt het onderzoek door te zoeken op '…….').
  - De volgende tekst is aangepast: '*Op de volgende website(s) vindt u meer informatie over het onderzoek. Na het onderzoek kan de website een samenvatting van de resultaten van dit onderzoek tonen (i.e. "U vindt het onderzoek door te zoeken op de website van PINCOR https://www.maastrichtuniversity.nl/nl/onderzoek/pincor-1)'.*

4. In paragraaf 13, we informeren uw huisarts: de tekst ''indien nodig kunnen we dan met uw revalidatiearts of huisarts contact opnemen over uw medische geschiedenis of de medicatie die u gebruikt'' verwijderen.
   - De volgende zin in paragraaf 13 is verwijderd: '*Dit is voor uw eigen veiligheid, indien nodig kunnen we dan met uw revalidatiearts of huisarts contact opnemen over uw medische geschiedenis of de medicatie die u gebruikt*'.
5. M.b.t. bijlage A: alleen de gegevens van de uitvoerend onderzoeker en de hoofdonderzoekers (prof. Verbunt en drs. Wiertz) vermelden; de contactgegevens van de hoofdonderzoeker nog toevoegen (e-mailadres en telefoonnummer). Verder: zowel de contactgegevens van de FG van Maastricht University als van de FG van Adelante vermelden. Tot slot m.b.t. informatie over de rechten van de deelnemer: onder het kopje ''Voor meer informatie over uw rechten'' de contactgegevens (inclusief website) van de verantwoordelijke(n) voor de verwerking van persoonsgegevens (i.e. van Maastricht University en van Adelante) vermelden.

- De contactgegeven van een groot deel van de onderzoeksgroep zijn verwijderd. De contactgegevens van de Functionaris Gegevensbescherming van zowel Maastricht University en Adelante zijn toegevoegd.

6. M.b.t. de toestemmingsverklaring: de passage ''Ik geef toestemming voor het verzamelen, gebruiken en bewaren van mijn persoonsgegevens zodat de onderzoeksvraag van dit onderzoek beantwoord kan worden'' vervangend door "Ik geef de onderzoekers toestemming om mijn gegevens te verzamelen en gebruiken. De onderzoekers doen dit alleen om de onderzoeksvraag van dit onderzoek te beantwoorden''. Verder: de tekst ''De proefpersoon krijgt een volledige informatiebrief mee, samen met een getekende versie van het toestemmingsformulier'' verwijderen. De toestemmingsverklaring dient op locatie door de onderzoeker en de deelnemer getekend te worden in elkaars aanwezigheid.
    - De verschillende teksten in de toestemmingsverklaring zijn aangepast.
7. Het NL-nummer van de studie nog opnemen in de PIF en in de toestemmingsverklaring (bijv. na de titel van het onderzoek).
    - NL nummer van de studie (NL83848.068.23) is toegevoegd in de PiF en in de toestemmingsverklaring.

<u>Met betrekking tot de PIF voor de zorgverleners:</u>

1. Algemeen: U wilt zorgverleners vragen om te participeren in een focusgroep gesprek. De voorliggende PIF is veel te uitgebreid. Een veel kortere PIF volstaat. Van belang is dat in de PIF wordt uitgelegd waarom de zorgverlener voor het focusgesprek wordt uitgenodigd, wat het doel is van het focusgesprek, waar het gesprek plaatsvindt, hoeveel tijd het gesprek kost, hoe er wordt omgegaan met de gegevens, dat er een vergoeding voor deelname is en waar de zorgverlener met eventuele vragen terecht kan.
    - We danken de METC voor de verschillende feedback punten rondom de PIF voor de zorgverleners. De PIF voor de zorgverleners is ingekort. Zie de track-changes in de PIF voor de verschillende aanpassingen.
2. U beschrijft in de PIF (paragraaf 4 en paragraaf 10) dat u (persoons)gegevens zoals naam, geboortedatum/leeftijd, e-mailadres, geslacht en informatie over de werkzaamheden (binnen welke discipline werkzaam bent en jaren werkervaring) wilt verzamelen. Waarom is het nodig om te vragen naar leeftijd/geboortedatum en geslacht van de zorgverlener? Deze gegevens lijken niet nodig voor het beantwoorden van de onderzoeksvragen. Alleen noodzakelijke persoonsgegevens mogen verzameld worden.
    - Er is besloten om geen informatie meer te verzamelen zoals leeftijd, geboortedatum en geslacht. De gegevens die verzameld zullen worden zijn naam en informatie over werkzaamheden. Zie ook de aanpassingen middels de track-changes in de PIF.
3. De koptekst van de informatiebrief voor zorgverleners vereist aanpassing; 'proefpersoneninformatie voor menen die corona hebben gehad' is niet correct.
    - De tekst in de koptekst is aangepast naar: 'Proefpersoneninformatie voor zorgverleners die patiënten met post-COVID syndroom behandelen - PINCOR'.
4. Voor opmerkingen m.b.t. paragraaf 10: zie punt 3 bij de vragen en opmerkingen m.b.t. de PIF voor de patiënten.
    - De verschillende aanpassingen zijn gedaan in de PIF voor zorgverleners. Zie de verschillende track-changes.
5. Voor opmerkingen m.b.t. bijlage A: zie punt 5 bij de vragen en opmerkingen m.b.t. de PIF voor de patiënten.
    - De verschillende opmerkingen zijn verwerkt in Bijlage A, zie track-changes in de PIF voor zorgverleners.

6. M.b.t. de toestemmingsverklaring: de passage ''Ik geef toestemming voor het verzamelen, gebruiken en bewaren van mijn persoonsgegevens zodat de onderzoeksvraag van dit onderzoek beantwoord kan worden'' vervangend door "Ik geef de onderzoekers toestemming om mijn gegevens te verzamelen en gebruiken. De onderzoekers doen dit alleen om de onderzoeksvraag van dit onderzoek te beantwoorden''. Verder: de tekst ''De proefpersoon krijgt een volledige informatiebrief mee, samen met een getekende versie van het toestemmingsformulier'' verwijderen. De toestemmingsverklaring dient op locatie door de onderzoeker en de deelnemer getekend te worden in elkaars aanwezigheid.
   - De opmerkingen zijn verwerkt in de toestemmingsverklaring, zie de track-changes. In de inleiding is de volgende tekst aangepast: '*Als u wilt meedoen, zal er een afspraak worden gepland om het toestemmingsformulier dat u vindt in bijlage B te ondertekenen op de revalidatie afdeling van het MUMC+*'.
7. Het NL-nummer van de studie nog opnemen in de PIF en in de toestemmingsverklaring (bijv. na de titel van het onderzoek)
   - Het NL-nummer van de studie (NL83848.068.23) is toegevoegd aan de PIF. Zowel na de titel als bij de toestemmingsverklaring.

Na ontvangst van uw reactie en een exemplaar van de aangepaste documenten zal de commissie zo spoedig mogelijk uw reactie bespreken. Op http://toetsingonline.ccmo.nl kunt u de stand van zaken van uw beoordeling volgen. De commissie verwacht dat u binnen drie maanden na dagtekening reageert. Na deze termijn zal zij uw voorstel seponeren, tenzij u een met redenen omkleed verzoek indient om de behandeling aan te houden.

Met vriendelijke groet, namens de METC azM/UM,

mevr. dr. E.C.H. van den Ham,
ambtelijk secretaris

prof. dr. H.C. Schouten,
vicevoorzitter

Kopieën: Prof. dr. J.A. Verbunt, Department of Rehabilitation Medicine, Maastricht University
Dr. D. Ummels, Department of Rehabilitation Medicine, Maastricht University

Bijlage 1: Administratieve aspecten

Bijlage 1: Administratieve aspecten

Met betrekking tot het onderzoeksprotocol:

1. Het versienummer en de versiedatum (graag een actuele datum aanhouden) in de voettekst graag in overeenstemming brengen met versienummer en datum in de tabel op pagina 2 van het protocol.
    a. Versienummer en versiedatum zijn aangepast in het C1-protocol
2. Protocol (versie clean) op blz. 31, paragraaf 8.1: in regel 2 verwijderen " with care-as-usual".
    a. De tekst is aangepast naar: '*The primary objective of the study is to test the effectiveness of a 12-week personalized interdisciplinary rehabilitation treatment in secondary care and to evaluate changes in the recovery of participation levels and quality of life in patients with post-COVID syndrome*'.

Met betrekking tot het ABR-formulier:

1. D10: graag wijzigen in ''ja'' en de vraag verder invullen.
    a. D10 is gewijzigd in ja en verdere toelichting is gegeven in D10a en D10b.
    b. D10a: "De proefpersonen worden geïnformeerd over de mogelijkheid van deelname aan het onderzoek door de behandelend revalidatie arts, welke eveneens onderdeel zijn van het onderzoeksteam."
    c. D10b: "Er is tot op heden nog geen effectiviteit onderzocht van de revalidatiebehandeling van patiënten met Post-Covid Syndroom, derhalve worden deze dan ook gevraagd om deel te nemen. De belangen van de proefpersoon worden gewaarborgd omdat de behandelend revalidatie arts alleen maar informeert over de studie. Het onderzoeksteam benaderd de proefpersoon voor deelname, dit gebeurt niet door een behandelaar. Ongeacht wel/geen deelname aan het onderzoek blijft de behandeling hetzelfde."
2. E2: de totale duur van het onderzoek voor de individuele deelnemer bedraagt ongeveer 7 maanden (zie ook de informatie in de PIF). Dit graag aanpassen.
    a. We hebben de totale duur aangepast naar 7 maanden. Daarbij hebben we de totale tijdsbeslag van de belasting van het onderzoek ook aangepast naar 7.5 uur i.v.m. enkele extra dagboekjes.
3. E9a: hier graag nog toevoegen dat de revalidatiebehandeling die deelnemers tijdens het onderzoek ondergaan niet anders is dan de revalidatiebehandeling die zij zouden ontvangen als zij niet aan het onderzoek meegedaan zouden hebben.
    a. We hebben een zin toegevoegd aan dit onderdeel. "*Ongeacht wel/geen deelname aan het onderzoek blijft de revalidatiebehandeling hetzelfde*."
4. E12: wijzigen in ''ja''. Bij deelname aan de studie start de revalidatiebehandeling 3-5 weken later dan bij niet deelnemen aan de studie.
    a. We hebben dit niet gewijzigd in "ja", zie ook eerdere reactie in deze brief. De reguliere wachtlijst voor de revalidatiebehandeling is gemiddeld 4-6 weken. De extra tijd die proefpersonen nodig hebben voor het invullen van de baseline metingen is dus niet nadelig voor de instroom in de revalidatiebehandeling.
5. F1: aanpassen conform de opmerkingen bij het protocol.
    a. Aangepast volgens protocol, extra zin toegevoegd m.b.t. het tekenen van de informed consent.
6. F4b en F4c: aanpassen conform de opmerkingen bij het protocol.
    a. Aangepast volgens protocol.

Met betrekking tot het onderzoekscontract tussen Maastricht University en Adelante:
Het voorliggende contract is akkoord wat betreft de bepalingen ten aanzien van voortijdige beëindigingen en publicatie. Een versienummer en versiedatum ontbreken nog in de voettekst van het contract. De commissie heeft een versie van het contract nodig waarop een versienummer en versiedatum zijn opgenomen om een positief besluit uit te kunnen brengen.. Een volledig getekend contract kan eventueel ook na afgifte van het besluit aan de commissie worden voorgelegd; het onderzoek kan echter pas van start gaan nadat de commissie de volledig getekende versie van het contract ter kennisgeving heeft aangenomen.

- Een versienummer en datum is toegevoegd aan het onderzoek contract.
